# Effectiveness and Safety of Acupuncture for Vascular Cognitive Impairment: A Systematic Review and Meta-Analysis

**DOI:** 10.3389/fnagi.2021.692508

**Published:** 2021-08-06

**Authors:** Xin-Tong Su, Ning Sun, Na Zhang, Li-Qiong Wang, Xuan Zou, Jin-Ling Li, Jing-Wen Yang, Guang-Xia Shi, Cun-Zhi Liu

**Affiliations:** ^1^International Acupuncture and Moxibustion Innovation Institute, School of Acupuncture-Moxibustion and Tuina, Beijing University of Chinese Medicine, Beijing, China; ^2^Traditional Chinese Medicine (TCM) in the Prevention and Rehabilitation of Stroke Task Force, World Stroke Organization, Geneva, Switzerland; ^3^Acupuncture and Tuina School/The 3rd Teaching Hospital, Chengdu University of Traditional Chinese Medicine, Chengdu, China; ^4^School of Acupuncture-Moxibustion and Tuina, Shandong University of Chinese Medicine, Jinan, China

**Keywords:** Hasegawa's Dementia Scale, Montreal Cognitive Assessment, Alzheimer's Disease Assessment Scale-Cognitive Subscale, Barthel ADL Index, Functional Activities Questionnaire

## Abstract

**Background:** Acupuncture may be a promising complementary therapy for vascular cognitive impairment (VCI) and has been extensively applied in China. However, its potential effects remain uncertain, and the clinical findings are inconsistent. This review aimed to systematically appraise the overall effectiveness and safety of acupuncture in treating VCI.

**Methods:** To investigate the effects of acupuncture on VCI from inception to February 28, 2021 using randomized clinical trials (RCTs), seven electro-databases [Cochrane Central Register of Controlled Trials (CENTRAL), PubMed, Embase, China National Knowledge Infrastructure (CNKI), Chinese Biomedical Literature Database (CBM), VIP, and Wanfang] were searched. Two independent investigators identified the eligible RCTs and extracted data into predesigned forms. The risk of bias (ROB) within each individual trial was evaluated using the Cochrane Collaboration's tool. Meta-analyses were conducted for calculating comparative effects in the RevMan software (version 5.3). The strength of attained evidence was rated using the online GRADEpro approach.

**Results:** A total of 48 RCTs involving 3,778 patients with VCI were included. The pooled data demonstrated that acupuncture was more beneficial for a global cognitive function [mean difference (MD) 1.86, 95% CI 1.19–2.54, *p* < 0.01] and activities of daily living (MD −3.08, 95% CI −4.81 to −1.35, *p* < 0.01) compared with western medicine (WM). The favorable results were also observed when acupuncture was combined with WM (MD 2.37, 95% CI 1.6–3.14, *p* < 0.01) or usual care (UC, MD 4.4, 95% CI 1.61–7.19, *p* = 0.002) in comparison with the corresponding control conditions. Meanwhile, the subgroup analysis did not indicate a statistical effect difference between manual acupuncture (MA) and electroacupuncture (EA) (inter-group *I*^2^ < 50% and *p* > 0.1) when comparing acupuncture with WM. There were no significant differences in the occurrence of adverse events (AEs) between the acupuncture group and the control group (*p* > 0.05). Owing to the poor methodological quality and considerable heterogeneity among studies, the certainty of the evidence was low or very low.

**Conclusions:** This review suggests that acupuncture as a monotherapy or an adjuvant therapy may play a positive role in improving the cognition and daily performance of VCI patients associated with few side effects. The difference in styles may not significantly influence its effectiveness. More rigorously designed and preregistered RCTs are highly desirable to verify the therapeutic benefits and determine an optimal acupuncture paradigm. The methodological and reporting quality of future researches should be enhanced by adhering to authoritative standardized statements.

**Systematic Review Registration:** [PROSPERO], identifier [No. CRD42017071820].

## Introduction

Next to Alzheimer's disease (AD), vascular cognitive impairment (VCI) is the second most frequent form of cognitive disorders, encompassing the full spectrum ranging from vascular subjective cognitive decline to vascular dementia, which is mainly caused by the diseased cerebral vasculature (O'Brien and Thomas, 2015; van der Flier et al., 2018). The prevalence of VCI is estimated at 1–1.5% in the global population above 65 years old (Jia et al., 2014; Rizzi et al., 2014), whereas the incidence of VCI increases with age, with the risk approximately doubling every 5.3 years, just slightly lower than that of AD (Jorm and Jolley, 1998). Meanwhile, VCI is also a serious challenge to healthcare providers and policymakers as the concomitant of the aging issue, which carries a heavy financial burden ranging from $17,000 to $55,200 per patient (Quentin et al., 2010; Zhou et al., 2019). The major underlying pathophysiology of VCI incorporates the interactions between vascular etiology, cerebral tissue dysfunction, white matter lesions, atrophy, and host factors such as age and education (Skrobot et al., 2016; Dichgans and Leys, 2017). Even though VCI is considered clinically and pathologically different from AD, VCI oftentimes coexists along with AD among older adults in the clinic (concurrent mixed dementia; Cechetto et al., 2008; Levine and Langa, 2011). In comparison to AD, the progress toward seeking available treatments for VCI has proven to be even more elusive and sluggish (O'Brien and Thomas, 2015). So far, the regulatory bodies and guideline groups have not approved any licensed drugs for effective disease modification in VCI. The present predominant strategy emphasizes symptomatic improvement and optimization of the quality of life for patients with VCI (Moniz-Cook et al., 2008; O'Brien and Thomas, 2015; National Collaborating Centre for Mental Health, 2018). More feasible therapeutic options for VCI are urgently needed.

Acupuncture, as an essential modality of traditional Chinese medicine (TCM), has been commonly practiced in the prevention and treatment of various diseases for millennia (Ulett et al., 1998). In recent decades, it receives increased attention from both the public and health professionals worldwide, even arousing the interest of major academic medical centers, especially for chronic disorders, which are difficult to be managed with conventional therapies (NIH Consensus Conference, 1998; Burke et al., 2006; World Health Organization, 2013). There are many categories of acupuncture approaches such as manual acupuncture (MA), electroacupuncture (EA), and scalp acupuncture (SA), which have turned out to be relatively less expensive with few adverse effects (Witt et al., 2009). As a non-pharmacological intervention with the intention to make patients recover to the postulated equilibrium state prior to illness (Endres et al., 2007), acupuncture has already been extensively used for VCI in plenty of Chinese medical institutions (Peng et al., 2007; Su et al., 2020a). A considerable number of emerging clinical trials demonstrated that acupuncture can serve as a promising treatment in improving the global cognitive status of patients with VCI (Chen et al., 2016, 2020; Yang et al., 2019). Meanwhile, various preclinical studies have also been conducted to explore the potential mechanisms of acupuncture *via* using the VCI animal model (Ye et al., 2017a; Du et al., 2018; Xiao et al., 2018). There may be multiple factors contributing to the neuroprotective effects of acupuncture, which can defer the pathological process of VCI. The underlying mechanisms of acupuncture are possibly reflected in protecting the neurons from oxidative stress, apoptosis, and neuroinflammation and in regulating glucose metabolism and neurotransmitters. In addition, another possible mechanism supporting the beneficial effect of acupuncture on VCI may be the enhancement of synaptic plasticity and blood vessel function (Ye et al., 2017b).

So far, systematic reviews (SRs) of acupuncture for VCI are relatively scant, whereas most of the existing randomized control trials (RCTs) are limited by the small sample size and study design flaws, which may bring about controversial results and cannot provide adequate evidence for further clinical applications. There was a Cochrane SR intending to appraise the efficacy and safety of acupuncture in treating VCI, which was firstly published in 2007 and updated in 2011 (Peng et al., 2007). However, due to its overcritical literature inclusion criteria, this SR did not include any RCTs and reached no valuable conclusion finally. Another SR published in 2017 only assessed the quality of reports concerning RCTs of SA for the treatment of VCI but did not synthesize the clinical outcomes (You et al., 2017). Given that there has been a further increase in newly published studies over the recent years since these two SRs have been published, it is of a strong necessity for us to conduct an updated SR and meta-analysis to re-evaluate its clinical benefits and safety.

## Materials and Methods

The review was undertaken and reported in accordance with the preferred reporting items for systematic reviews and meta-analyses (PRISMA) statement (Moher et al., 2009) (see Supplementary Table 1). A detailed prospective protocol of this study was registered at PROSPERO (Center for Reviews and Dissemination, University of York, No. CRD42017071820) before formal commencement. There was no deviation from the approved protocol.

### Literature Search Strategy and Study Selection Criteria

The following seven electro-databases were searched for the relevant RCTs published from database inception to February 28, 2021: Cochrane Central Register of Controlled Trials (CENTRAL), PubMed, Embase, China National Knowledge Infrastructure (CNKI), Chinese Biomedical Literature Database (CBM), VIP database, and Wanfang database. Additionally, the bibliographic lists of the identified publications and ambiguous literatures were manually searched. Five prestigious TCM-relevant journals were searched as a supplement resource. Two independent reviewers (X-TS and NS) comprehensively searched and filtrated the eligible studies. The predesigned search syntax used in PubMed as an example can be found in the protocol (Ye et al., 2017c). The equivalent search terms were applied in different databases. We decided not to include gray literatures to guarantee the quality of the further analyzed studies.

The titles and abstracts of all initially identified articles were screened and examined according to the patient, intervention, control, outcome, and study design (PICOS) selection principle after a duplication check, a full-text review for the detailed features was performed if necessary. Disagreements regarding study eligibility were resolved through a consensus meeting with the corresponding author. The study selection criteria were the following PICOS elements: (1) Patients met a diagnosis of VCI based on any established, clear, and validated diagnostic definitions including the 4th edition of Diagnostic and Statistical Manual of Mental Disorders (DSM-IV) (American Psychiatric Association, 1994), National Institute of Neurological Disorders and Stroke (NINDS-AIREN) (Roman et al., 1993), the 10th revision of International Classification of Diseases (ICD-10) (World Health Organization, 1992), and the diagnostic criteria for vascular behavioral and cognitive disorders (VASCO) (Sachdev et al., 2014). The onset of cognitive impairment was required to be definitely and causally linked with the presence of cerebrovascular pathogenesis. Additionally, we excluded the studies that did not apply an imaging technique/brain scan to differentiate other subtype-specific diagnoses of cognitive impairment. (2) Regarding acupuncture in the experimental groups, MA, EA, SA, auricular acupuncture, fire needling, and warm needling were included without any limitation on the type. RCTs were accepted if acupuncture was applied as a sole therapy or as an adjunct to the same interventions in the control groups. (3) To evaluate the true effectiveness of acupuncture for VCI, we limited the interventions in the control groups to sham acupuncture (mimicking true acupuncture but deviating from the TCM theory, such as placebo acupuncture at non-acupoints) or no treatment. Meanwhile, western medicine (WM), usual care (UC), or other conventional treatments like cognitive rehabilitation were also included. We did not consider RCTs that compared different acupoints or acupuncture techniques, neither study exploring the disparity between acupuncture with other TCM therapies. (4) The primary outcomes focused on a global cognitive function and behavioral disturbances, which could be measured by validated and standardized scales such as the mini mental state examination (MMSE) score, the Hasegawa's Dementia Scale (HDS), the Montreal Cognitive Assessment (MoCA) score, and the Alezheimer's Disease Assessment Scale-cognitive subscale (ADAS-cog). The secondary outcomes included general skill levels regarding daily functioning and a dependent degree to caregivers and therapeutic safety. The former could be assessed by the Activities of Daily Living (ADL) Scale, the Barthel ADL Index (BI), and the Functional Activities Questionnaire (FAQ). Therapeutic safety was reported with the incidence and specific types of adverse events (AEs). (5) RCTs regardless of publication language restrictions were included, whereas non-RCTs, uncontrolled trials, and protocols for RCTs were excluded. We defined the studies as RCTs if the allocation of participants was generated by specific methods of random sequence. If ambiguous or no further description of the method was provided, we contacted the corresponding authors *via* telephone or e-mail to make certain of the details.

### Data Collection Process and Study Quality Assessment

Microsoft Excel was utilized to compile an electronic data extraction form and manage the information extracted from the eligible articles, including the general characteristics of the study, eligibility criteria, participant demographics, interventions, weight-related outcomes, and AEs. All data were extracted and cross-checked by the investigators to ensure accuracy (NZ and J-LL). Corresponding authors were contacted and requested to clarify ambiguities and provide missing information *via* telephone or e-mail if available data could not be acquired directly from the articles. As to trials with multiple therapy groups, irrelevant data from other arms were not extracted for analysis.

The methodological quality of each included study was assessed with an aid of the Cochrane Collaboration's risk of bias (ROB) tool. There were seven specific items appraised and recorded separately in a structured form by two reviewers (NZ and XZ). Any discrepancies concerning the assessments were resolved through the discussion with a methodological researcher (L-QW). The ROB could be categorized into three levels based on the Cochrane Handbook: low, unclear, and high. In addition, given that most of the acupuncture studies were published in Chinese journals, we also evaluated the reporting quality of the included RCTs with reference to the internationally recognized Consolidated Standards for Reporting of Trials (CONSORT) statement (Schulz et al., 2010), and Standards for Reporting Interventions in Controlled Trials of Acupuncture (STRICTA) reporting guideline (MacPherson and Jobst, 2010). The reporting percentage of each item of the corresponding norms was calculated and presented.

### Data Synthesis and Statistical Methods

The RevMan software 5.3 (Cochrane Collaboration, London, UK) was used for all data analyses (Higgins et al., 2019). Heterogeneity among studies was examined and quantified with the *Q*-test (*p*-value) and *I*^2^ statistic (percentage value). If the heterogeneity was significant (*p* < 0.1, *I*^2^ > 50%), a random effect (RE) model was chosen to pool the data, and if there was acceptable heterogeneity (*p* ≥ 0.1, *I*^2^ ≤ 50%), a fixed-effect (FE) model was used. The mean difference (MD) or standardized mean difference (SMD) with 95% CI was calculated for continuous outcomes, whereas the risk ratio (RR) with 95% CI was measured for dichotomous outcomes. The *Z*-test was conducted to determine the significance of the pooled results, and a statistically significant difference was set as two-sided value of *p* < 0.05. We placed the included studies into categories according to the type of control groups, which were WM, UC, and no treatment. The subgroup analysis was performed to interpret the possible heterogeneity with the stratified factor of different acupuncture types. Sensitivity analysis was conducted to verify the robustness of the meta-analysis results and explore the potential sources of heterogeneity after the omission of each individual study from the original analysis. Funnel plots were generated to estimate the reporting biases for the outcomes of more than 10 studies (Sterne et al., 2000). The overall quality and certainty of the evidence for therapeutic effect estimation were rated by the online GRADEpro GDT approach at www.gradepro.org (Guyatt et al., 2008).

## Results

### Study Characteristics

**Figure 1:**
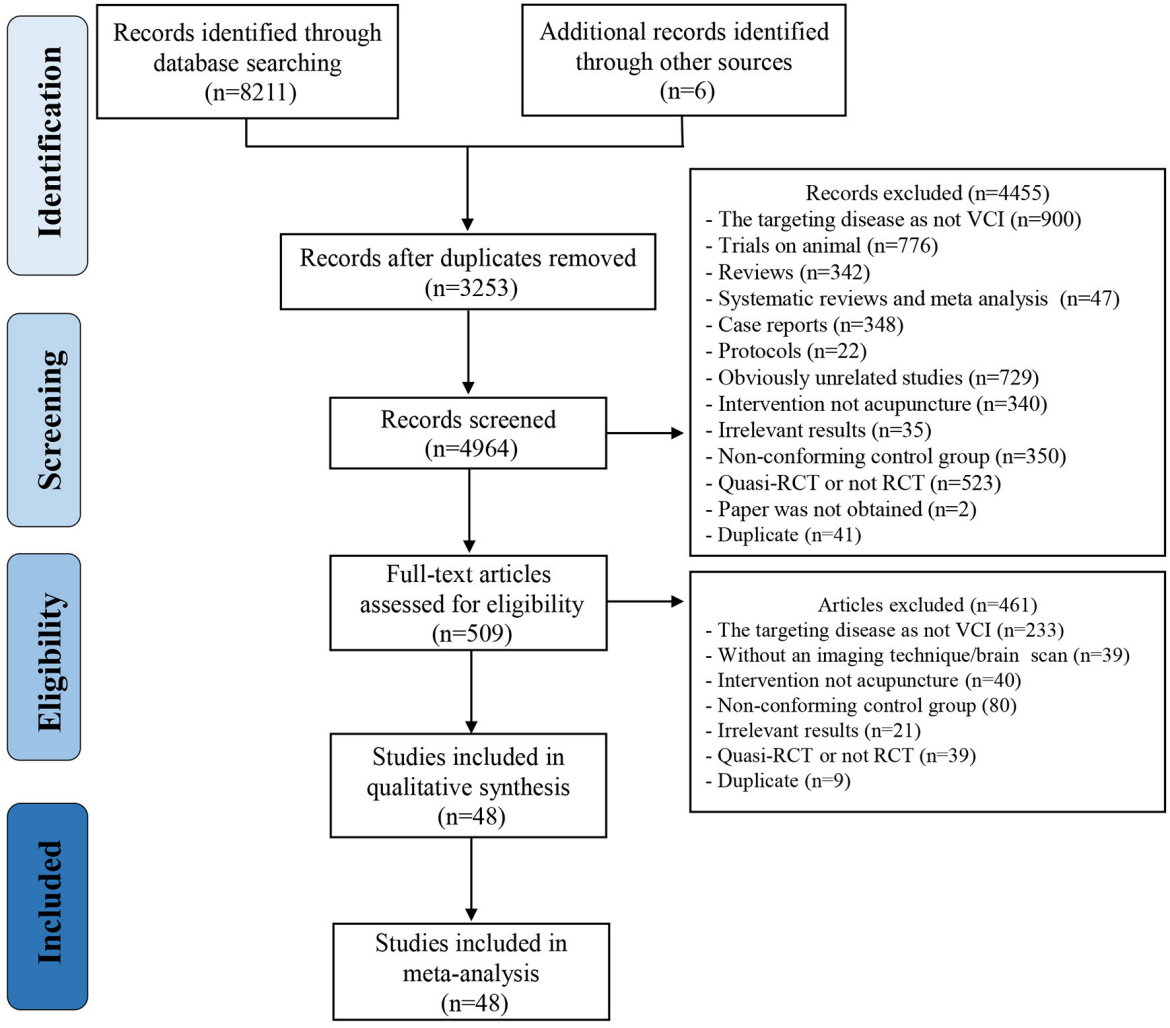
Preferred reporting items for systematic reviews and meta-analyses (PRISMA) flow diagram of literature inclusion process.

An initial search yielded 8,211 citations from the seven electronic databases in total, and six records were identified through additional sources. After checking duplicates and screening titles and abstracts, 509 full-texts were further read. As a result, 48 RCTs, published from 1992 to 2020, involving 3,778 patients with VCI met the eligible criteria and were synthesized in the final quantitative meta-analysis (Huang et al., 1992; Mo et al., 2000a,b; Zhao et al., 2000, 2009; Jiang et al., 2003, 2019; Liu et al., 2006; Niu and Liu, 2007; Chu et al., 2008; Liu Y. et al., 2008; Liu Z. B. et al., 2008; Zhang et al., 2008, 2015, 2019; Chen et al., 2009, 2015, 2020; Li et al., 2009, 2014, 2019; Meng et al., 2009, 2020; Shi et al., 2009; Yin et al., 2011; Lin et al., 2012; Li P. et al., 2012; Li S. et al., 2012; Li W. et al., 2012; Teng and Lai, 2012; Pan and Ai, 2013; Zhao and Xu, 2013; Cao et al., 2014; Li and Jiao, 2014; Cui et al., 2015; Luo et al., 2015; Yang et al., 2016, 2019; Tan et al., 2017; Wang and Wang, 2017a,b,c; Cheng et al., 2018; Feng et al., 2019; Hu et al., 2019; Qu et al., 2020; Xiong et al., 2020; Yao R. et al., 2020). Of all these studies performed in China, six articles were published in English. The PRISMA flow diagram of the literature inclusion process is presented in Figure 1.

In these studies, VCI was diagnosed according to the following criteria: DSM-IV (62.5%), NINDS-AIREN (22.9%), VASCO (12.5%), and ICD-10 (2%). The proportion of men across the studies varied from 35 to 75% with an average age of 65.4 years. The sample sizes fell within the scope of 36–270. 48 studies employed 51 comparison pairs in total, wherein 3 RCTs applied a three-arm parallel clinical design (Liu et al., 2006; Zhang et al., 2008; Zhao et al., 2009). Of these comparison pairs, 27 compared acupuncture with WM, 17 compared acupuncture plus WM with WM alone, and 7 compared acupuncture plus UC with UC alone. 13 studies reported dropouts together with the provided reasons, which ranged from 1 to 29 participants. 21 RCTs did not report funding sources, whereas the other 27 studies were funded by governmental grants. Table 1 summarizes the detailed information of the 48 included RCTs.

**Table 1:** Characteristics of the included studies.

**Study**	**Diagnostic criteria**	**Mean age (y)**	**Men (%)**	**Sample size (T/C)**	**Disease duration (T/C)**	**Acupuncture**					**Control type**	**Outcomes**	**Measurement time points**
						**Style**	**Course (d)**	**Retention (min)**	**Frequency (t/w)**	***De-qi* response**			
Cao et al. (2014)	DSM-IV	65	60	40/40	NR	MA	28	30	6	√	Nicergoline	HDS	4w
Chen et al. (2020)	VASCO	64.9	59	28/28	NR	MA (SA) + Donepezil	28	30	12	NR	Donepezil	MoCA, ADLS	4w
Chen et al. (2009)	DSM-IV	68.4	50	33/33	26.5/26.1m	MA	30	30	7	√	Monosialotetrahexosylganglioside Sodium	HDS	30d
Chen et al. (2015)	DSM-IV	66	55	38/37	3.7/4.1y	MA + Oxiracetam	30	30	7	NR	Oxiracetam	MMSE	30d
Cheng et al. (2018)	NINDS-AIREN	67	47	25/26	NR	MA	90	NR	6	√	Donepezil	MMSE	90d
Chu et al. (2008)	NINDS-AIREN	67.9	58	32/33	4.9/5.2m	MA (SA) + Duxil	56	600	5	√	Duxil	MMSE, HDS, ADLS	8w
Cui et al. (2015)	DSM-IV	67	53	30/30	4.9/5m	MA + Nimodipine	28	40	6	√	Nimodipine	MMSE	4w
Feng et al. (2019)	NINDS-AIREN	64.1	67	47/47	7.6/6.4m	MA + Butylphthalide	56	30	7	NR	Butylphthalide	MMSE	8w
Hu et al. (2019)	DSM-IV	67.5	53	44/44	2.5/2.8y	MA (SA) + Oxiracetam	56	NR	6	√	Oxiracetam	MMSE, BI	8w
Huang et al. (1992)	ICD-10	NR	75	18/18	NR	MA + Citicoline	15	NR	7	NR	Citicoline	HDS	15d
Jiang et al. (2003)	DSM-IV	NR	59	23/23	NR	EA	28	30	5	√	Nimodipine	HDS, BI	4w
Jiang et al. (2019)	VASCO	58	57	30/30	NR	EA + Usual care	56	45	5	√	Usual care	MMSE, MoCA, BI	8w
Li et al. (2019)	DSM-IV	67.2	50	40/40	89.4/90.6d	MA + Donepezil	42	30	5	√	Donepezil	MMSE, MoCA, BI	6w
Li and Jiao (2014)	DSM-IV	66.8	54	50/50	32.3/30.7	MA	84	30	5	NR	Oxiracetam	MMSE, ADLS, ADAS-cog	12w
Li P. et al. (2012)	NINDS-AIREN	NR	56	50/50	NR	MA	28	40	6	√	Nimodipine	MMSE, HDS, BI	4w
Li S. et al. (2012)	DSM-IV	63	51	40/40	10.5/10.8y	MA (SA)	35	30	5.6	NR	Citicoline	MMSE, BI	35d
Li et al. (2014)	NINDS-AIREN	64	51	30/30	10.7/10.5y	MA	14	45	7	NR	Aniracetam	MMSE, BI	14d
Li W. et al. (2012)	NINDS-AIREN	68.7	58	50/50	13.3/12.3m	EA + Nimodipine	90	30	5	√	Nimodipine	MMSE, HDS, BI	90d
Li et al. (2009)	DSM-IV	62.8	57	30/30	NR	MA	70	30	6	√	Duxil	HDS	10w
Lin et al. (2012)	DSM-IV	64	60	20/20	NR	EA	28	30	6	√	Oxiracetam	MMSE, ADLS	4w
Liu et al. (2006)	DSM-IV	69.5	NR	20/20/20	NR	MA + Nimodipine MA	32	45	6.5	√	Nimodipine	HDS, BI, MMSE	30d
Liu Y. et al. (2008)	DSM-IV	65	64	54/54	NR	MA (SA)	35	30	6	NR	Nimodipine	MMSE, HDS, ADLS	30d
Liu Z. B. et al. (2008)	DSM-IV	68	52	30/30	5.7/4.7m	EA	70	NR	5	√	Duxil	MMSE, HDS, FAQ	10w
Luo et al. (2015)	NINDS-AIREN	71.7	45	36/36	1.3/1.3y	MA	56	30	3.5	NR	Donepezil hydrochloride	MMSE	8w
Meng et al. (2020)	VASCO	65.4	56	61/61	NR	MA + Donepezil	28	30	6	√	Donepezil	MMSE, MoCA, BI	4w
Meng et al. (2009)	DSM-IV	66.9	58	30/30	NR	MA	30	40	7	√	Nimodipine	HDS, MMSE	30d
Mo et al. (2000a)	DSM-IV	65.8	63	30/30	NR	EA	42	30	5	√	Dihydroergotoxine mesylate	HDS, BI, FAQ	6w
Mo et al. (2000b)	DSM-IV	65.3	60	31/31	NR	EA	42	30	5	√	Dihydroergotoxine mesylate	HDS, BI, FAQ	6w
Niu and Liu (2007)	DSM-IV	67	38	30/30	8.6/7.7m	MA (SA)	70	360	5	NR	Duxil	HDS	10w
Pan and Ai (2013)	DSM-IV	NR	NR	30/30	NR	MA (SA) + Usual care	70	30	5	√	Usual care	MMSE, HDS	10w
Qu et al. (2020)	DSM-IV	65.9	60	30/30	2.42/2.98y	MA + Oxiracetam	28	30	12	√	Oxiracetam	MMSE, MoCA, BI	4w
Shi et al. (2009)	DSM-IV	62.4	54	31/31	15.8/15.8m	MA + Citicoline	56	0	6	NR	Citicoline	MMSE, ADLS	8w
Tan et al. (2017)	DSM-IV	66.7	60	30/30	72.5/81.4d	MA	30	30	7	√	Nimodipine	MMSE	30d
Teng and Lai (2012)	DSM-IV	66	62	25/28	2.1/1.9y	MA + Duxil	30	30	6	NR	Duxil	MMSE, BI	30d
Wang and Wang (2017a)	NINDS-AIREN	68.1	51	60/60	5.7/6.2y	MA + Usual care	28	20	6	√	Usual care	MMSE, HDS, BI	4w
Wang and Wang (2017b)	NINDS-AIREN	69.3	59	40/40	8.4/8.3y	MA + Usual care	36	30	7	√	Usual care	MMSE, BI	36d
Wang and Wang (2017c)	NINDS-AIREN	66.2	48	43/43	5.1/5.2y	MA + Usual care	90	20	7	√	Usual care	MMSE, BI	90d
Xiong et al. (2020)	VASCO	64.2	53	35/35	2.1/2.5m	MA (SA) + Usual care	56	240	6	NR	Usual care	MMSE, ADLS	8w
Yang et al. (2019)	VASCO	65.4	35	108/108	NR	MA	84	30	2	√	Citicoline	ADAS-cog, ADLS	90d
Yang et al. (2016)	DSM-IV	67.6	62	60/60	1.3/1.2y	MA + Nicergoline	84	NR	5	NR	Nicergoline	ADLS, ADAS-cog	12w
Yao R. et al. (2020)	VASCO	56	67	30/30	2.5/2.4m	MA (SA) + Usual care	28	NR	6	NR	Usual care	MMSE, MoCA	4w
Yin et al. (2011)	DSM-IV	62.6	58	30/30	1.6/1.6y	EA	84	30	5	√	Nimodipine	MMSE	12w
Zhang et al. (2008)	DSM-IV	64.5	65	90/90/90	NR	EA + Nimodipine EA	42	30	5	√	Nimodipine	MMSE, ADLS	6w
Zhang et al. (2015)	DSM-IV	NR	53	20/20	NR	MA	28	40	6	√	Nimodipine	MMSE, ADLS	4w
Zhang et al. (2019)	DSM-IV	58.4	43	41/41	1.42/1.36y	MA	56	20	12	√	Donepezil	MoCA, BI	8w
Zhao et al. (2000)	DSM-IV	61	54	36/32	6.5/6.5m	EA	30	20	7	√	Hydergine	MMSE	30d
Zhao et al. (2009)	DSM-IV	NR	NR	30/30/30	NR	EA + Nimodipine EA	42	30	5	√	Nimodipine	MMSE	6w
Zhao and Xu (2013)	NINDS-AIREN	63.8	72	30/30	1.4/1.4m	MA	70	30	6	√	Duxil	MMSE, ADLS	60d

*ADAS-cog, Alzheimer's Disease Assessment Scale-cognitive subscale; ADLS, Activities of Daily Living Scale; BI, Barthel Index; DSM-IV, 4th edition of Diagnostic and Statistical Manual of Mental Disorders; EA, electroacupuncture; FAQ, Functional Activities Questionnaire; HDS, Hasegawa's Dementia Scale; ICD-10, 10th revision of International Classification of Diseases; MA, manual acupuncture; MMSE, Mini Mental State Examination; MoCA, Montreal Cognitive Assessment; NINDS-AIREN, National Institute of Neurological Disorders and Stroke; NR, no report; SA, scalp acupuncture; VASCO, diagnostic criteria for vascular behavioral and cognitive disorders*.

### Acupuncture Patterns and Acupoint Selection

Manual acupuncture (58.3%) was the most commonly conducted acupuncture type, followed by EA (22.9%) and SA (18.6%). 18 RCTs chose a flexible method for acupoint selection, whereas the remaining ones established the fixed formulas. The flexible formulas of acupoints were supplemented with the principal acupoints and were determined by different disease phases, syndromes, and basic characteristics of individuals. Sufficient deep needling was required in all trials; meanwhile, most of the trials (68.8%) emphasized adequate stimulation to elicit *de-qi* sensation. The needle retention time was set from 0 to 600 min with a usual mode of 30 min (*n* = 27). The frequency of treatment varied from 2 to 14 sessions per week with a welcomed mode of 5–6 times per week (*n* = 32). Although the duration of treatment periods showed a considerable variation, which ranged from 14 to 90 days, more than 95% of studies (*n* = 46) preferred a long therapeutic course lasting for at least 4 weeks.

The top 10 frequently used acupoints were *Bai-hui* (62.5%), *Si-shen-cong* (52.1%), *Shen-ting* (43.8%), *Feng-chi* (27.1%), *Nei-guan* (27.1%), *Shui-gou* (22.9%), *Zu-san-li* (18.8%), *San-yin-jiao* (18.8%), *Tai-xi* (18.8%), and *Ben-shen* (14.6%). The essential acupoints for VCI treatment are mainly distributed on the craniofacial region even though the researchers adopted multifarious protocols of acupoint selection and combination. The acupoints chosen within individual studies are summarized in Table 2.

**Table 2:** Formulas of acupoint selection.

**Study**	**Style**	**Main acupoints**	**Flexibility**
Cao et al. (2014)	MA	*Shui-gou* (GV26), *Bai-hui* (GV20), *Si-shen-cong* (EX-HN1), *Feng-chi* (GB20), *Wan-gu* (GB12), *Tian-zhu* (BL10), *Nei-guan* (PC6), *Feng-long* (ST40), *San-yin-jiao* (SP6)	\
Chen et al. (2020)	SA	*Parietal region, Frontal region, Temporal region, Occipital region, Suboccipital region, Anterior parietal region, Item region*	\
Chen et al. (2009)	MA	*Nei-guan* (PC6), *Shui-gou* (GV26), *San-yin-jiao* (SP6), *Bai-hui* (GV20), *Si-shen-cong* (EX-HN1), *Gan-shu* (BL18), *Shen-shu* (BL23)	√
Chen et al. (2015)	MA	*Shen-shu* (BL23), *Tai-xi* (KI3), *Bai-hui* (GV20), *Si-shen-cong* (EX-HN1), *He-gu* (LI4), *Tai-chong* (LR3), *Pi-shu* (BL20), *Zu-san-li* (ST36)	\
Cheng et al. (2018)	MA	*Shen-ting* (GV24), *Bai-hui* (GV20), *Feng-fu* (GV16), *Da-zhui* (GV14)	√
Chu et al. (2008)	SA	*Ding-zhong-xian* (MS5), *E-zhong-xian* (MS1), *Ding-pang-xian-I* (MS8)	\
Cui et al. (2015)	MA	*Bai-hui* (GV20), *Feng-fu* (GV16), *Ya-men* (GV15), *Shen-ting* (GV24), *Shui-gou* (GV26), *Da-zhui* (GV14), *Zhi-yang* (GV9), *Yao-yang-guan* (GV3)	√
Feng et al. (2019)	MA	*Jian-shi* (PC5), *Nei-guan* (PC6), *Da-ling* (PC7), *Lao-gong* (PC8)	\
Hu et al. (2019)	SA	*Parietal region, Frontal region, Anterior parietal region*	√
Huang et al. (1992)	MA	*Shen-ting* (GV24), *Bai-hui* (GV20), *Feng-chi* (GB20), *Shen-men* (HT7), *Da-zhong* (KI14)	√
Jiang et al. (2003)	EA	*Shen-ting* (GV24), *Ben-shen* (GB13), *Si-shen-cong* (EX-HN1)	√
Jiang et al. (2019)	EA	*Bai-hui* (GV20), *Shen-ting* (GV24)	\
Li et al. (2019)	MA	*Shen-ting* (GV24), *Bai-hui* (GV20), *Si-shen-cong* (EX-HN1), *Qi-hai* (CV6), *Guan-yuan* (CV4), *He-gu* (LI4), *Zu-san-li* (ST36), *Tai-chong* (LR3)	\
Li and Jiao (2014)	MA	*Ya-men* (GV15), *Lao-gong* (PC8), *San-yin-jiao* (SP6), *Yong-quan* (KI1), *Tai-xi* (KI3), *Zhong-wan* (CV12), *Huan-tiao* (GB30), *Zu-san-li* (ST36), *He-gu* (LI4)	\
Li P. et al. (2012)	MA	*Bai-hui* (GV20), *Feng-fu* (GV16), *Ya-men* (GV15), *Shen-ting* (GV24), *Shui-gou* (GV26), *Da-zhui* (GV4), *Zhi-yang* (GV9), *Yao-yang-guan* (GV3), *Chang-qiang* (GV1)	√
Li S. et al. (2012)	SA	*E-zhong-xian* (MS1), *Ding-nie-qian-xie-xian* (MS6), *Ding-zhong-xian* (MS5), *Zhen-shang-zheng-zhong-xian* (MS12), *Zhen-shang-pang-xian* (MS13)	\
Li et al. (2014)	MA	*Bai-hui* (GV20), *Si-shen-cong* (EX-HN1), *Feng-chi* (GB20), *Feng-fu* (GV16), *He-gu* (LI4), *Shui-gou* (GV26), *Nei-guan* (PC6), *Zu-san-li* (ST36), *Tai-chong* (LR3)	\
Li W. et al. (2012)	EA	*Bai-hui* (GV20), *Shen-ting* (GV24), *Qu-cha* (BL4), *Si-shen-cong* (EX-HN1), *Feng-chi* (GB20), *Nei-guan* (PC6), *He-gu* (LI4), *Zu-san-li* (ST36), *San-yin-jiao* (SP6), *Tai-xi* (KI3), *Zhao-hai* (KI6)	√
Li et al. (2009)	MA	*Shen-ting* (GV24), *Shang-xing* (GV23), *Ben-shen* (GB13), *Tou-wei* (ST8), *Shuai-gu* (GB8), *Si-shen-cong* (EX-HN1), *Bai-hui* (GV20), *Feng-chi* (GB20), *Xuan-zhong* (GB39), *Tai-xi* (KI3), *San-yin-jiao* (SP6)	\
Lin et al. (2012)	EA	*Shen-ting* (GV24), *Tou-wei* (ST8), *Bai-hui* (GV20), *Si-shen-cong* (EX-HN1), *Nei-guan* (PC6), *San-yin-jiao* (SP6)	\
Liu et al. (2006)	MA	*Bai-hui* (GV20), *Si-shen-cong* (EX-HN1), *Feng-chi* (GB20), *Shui-gou* (GV26), *Qu-chi* (LI11), *Zu-san-li* (ST36), *Xuan-zhong* (GB39), *Tai-xi* (KI3)	\
Liu Y. et al. (2008)	SA	*Ding-nie-qian-xie-xian* (MS6), *Ding-nie-hou-xie-xian* (MS7)	\
Liu Z. B. et al. (2008)	EA	*Ying-xiang* (LI20), *Yang-bai* (GB14), *Yin-tang* (EX-HN3)	\
Luo et al. (2015)	MA	*Dan-zhong* (CV17), *Zhong-wan* (CV12), *Qi-hai* (CV6), *Xue-hai* (SP10), *Zu-san-li* (ST36), *Wai-guan* (TE5)	\
Meng et al. (2020)	MA	*Bai-hui* (GV20), *Shen-ting* (GV24), *Shui-gou* (GV26), *Yin-tang* (EX-HN3), *Feng-fu* (GV16), *Tai-xi* (KI3), *Tai-chong* (LR3), *Feng-long* (ST40), *Nei-guan* (PC6), *San-yin-jiao* (SP6)	\
Meng et al. (2009)	MA	*Bai-hui* (GV20), *Qiang-jian* (GV18), *Nao-hu* (GV17)	√
Mo et al. (2000a)	EA	*Si-shen-cong* (EX-HN1), *Nei-guan* (PC6), *Feng-chi* (GB20), *Ben-shen* (GB13)	√
Mo et al. (2000b)	EA	*Si-shen-cong* (EX-HN1), *Nei-guan* (PC6), *Feng-chi* (GB20), *Ben-shen* (GB13)	√
Niu and Liu (2007)	SA	*Shen-ting* (GV24), *Tou-lin-qi* (GB15), *Ben-shen* (GB13), *Tou-wei* (ST8), *Shuai-gu* (GB8), *Qu-bin* (GB7), *Si-shen-cong* (EX-HN1), *Bai-hui* (GV20), *Yu-zhen* (BL9)	\
Pan and Ai (2013)	SA	*Bai-hui* (GV20), *Shen-ting* (GV24), *Si-shen-cong* (EX-HN1), *Ben-shen* (GB13)	√
Qu et al. (2020)	MA	*Shui-gou* (GV26), *Nei-guan* (PC6), *San-yin-jiao* (SP6), *Si-shen-cong* (EX-HN1), *Xuan-zhong* (GB39), *Tai-xi* (KI3)	\
Shi et al. (2009)	MA	*Shao-shang* (LU11), *Shang-yang* (LI1), *Li-dui* (ST45), *Yin-bai* (SP1), *Shao-chong* (HT9), *Shao-ze* (SI1), *Zhi-yin* (BL67), *Yong-quan* (KI1), *Zhong-chong* (PC9), *Guan-chong* (TE1), *Zu-qiao-yin* (GB44), *Da-dun* (LR1)	\
Tan et al. (2017)	MA	*Bai-hui* (GV20), *Shen-ting* (GV24), *Shui-gou* (GV26), *Nei-guan* (PC6), *Da-ling* (PC7), *Lao-gong* (PC8), *Da-zhui* (GV4)	√
Teng and Lai (2012)	MA	*Feng-chi* (GB20), *Yi-ming* (EX-HN13), *Feng-fu* (GV16), *Bai-hui* (GV20), *Si-shen-cong* (EX-HN1)	\
Wang and Wang (2017a)	MA	*Bai-hui* (GV20), *Si-shen-cong* (EX-HN1)	\
Wang and Wang (2017b)	MA	*Bai-hui* (GV20), *Si-shen-cong* (EX-HN1)	\
Wang and Wang (2017c)	MA	*Ben-shen* (GB13), *Shen-ting* (GV24), *Si-shen-cong* (EX-HN1), *Shen-men* (HT7)	√
Xiong et al. (2020)	SA	*Bai-hui* (GV20), *Si-shen-cong* (EX-HN1), *Feng-chi* (GB20), *Shen-ting* (GV24)	\
Yang et al. (2019)	MA	*Zu-san-li* (ST36), *Xue-hai* (SP10), *Dan-zhong* (CV17), *Zhong-wan* (CV12), *Qi-hai* (CV6), *Bai-hui* (GV20), *Feng-fu* (GV16), *Xin-shu* (BL15), *Yi-xi* (BL45), *Tong-li* (HT5), *Zhao-hai* (KI6), *Tai-xi* (KI3), *Xuan-zhong* (GB39), *Feng-long* (ST40), *Nei-guan* (PC6), *Ge-shu* (BL17)	\
Yang et al. (2016)	MA	*Qi-hai* (CV6), *Zhong-wan* (CV12), *Dan-zhong* (CV17), *Xue-hai* (SP10), *Zu-san-li* (ST36), *Wai-guan* (TE5)	\
Yao R. et al. (2020)	SA	*Three temporal points*, Four *spiritual points*, Three *cerebral points*, Three *intelligent points*	√
Yin et al. (2011)	EA	*Si-shen-cong* (EX-HN1), *Shen-ting* (GV24), *Bai-hui* (GV20)	\
Zhang et al. (2008)	EA	*Si-shen-cong* (EX-HN1), *Bai-hui* (GV20), *Shen-ting* (GV24), *Feng-chi* (GB20)	\
Zhang et al. (2015)	MA	*Bai-hui* (GV20), *Feng-fu* (GV16), *Ya-men* (GV15), *Shen-ting* (GV24), *Shui-gou* (GV26), *Da-zhui* (GV4), *Zhi-yang* (GV9), *Yao-yang-guan* (GV3), *Chang-qiang* (GV1)	√
Zhang et al. (2019)	MA	*Shui-gou* (GV26), *Nei-guan* (PC6), *San-yin-jiao* (SP6), *Si-shen-cong* (EX-HN1), *Xuan-zhong* (GB39), *Tai-xi* (KI3)	\
Zhao et al. (2000)	EA	*Bai-hui* (GV20), *Shen-shu* (BL23), *Ge-shu* (BL17)	√
Zhao et al. (2009)	EA	*Si-shen-cong* (EX-HN1), *Bai-hui* (GV20), *Shen-ting* (GV24), *Feng-chi* (GB20)	\
Zhao and Xu (2013)	MA	*Bai-hui* (GV20), *Si-shen-cong* (EX-HN1), *Shen-ting* (GV24), *Shuai-gu* (GB8), *Tou-wei* (ST8), *Feng-chi* (GB20)	√

*MA, manual acupuncture; EA, electroacupuncture; SA, scalp acupuncture*.

### Methodological and Reporting Quality

**Figure 2:**
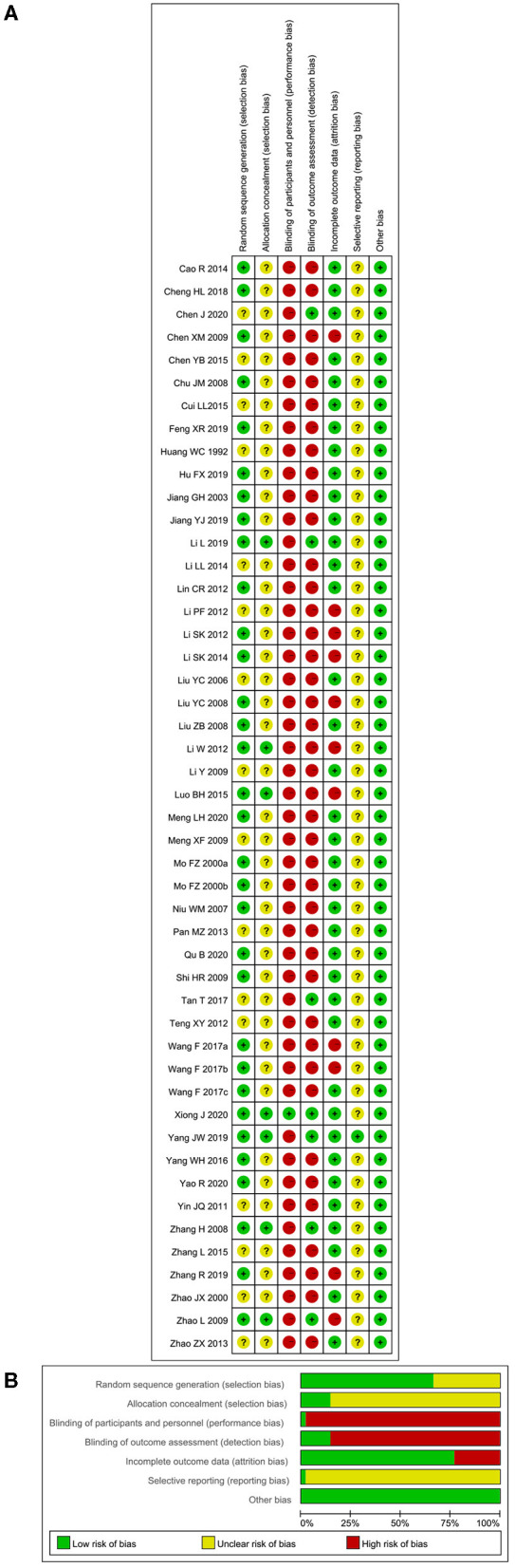
Assessment of risk of bias (ROB) using the Cochrane tool. **(A)** ROB graph and **(B)** ROB summary.

The methodological and reporting quality of the RCTs were universally unsatisfactory. We contacted the corresponding authors to make clear details of each bias if it was not described or if it was ambiguous. 32 RCTs appropriately reported their methods of sequence generation by means of a random number table or SAS statistical package, whereas the other 16 studies were rated as “unclear risk” because the articles only mentioned “randomization” and the corresponding authors did not provide further details. Only seven studies (14.6%) applied sealed and opaque envelopes to conceal allocation. Although there was inadequate information concerning allocation concealment, no baseline differences existed between the groups in these trials. More than 80% of the RCTs did not report the details about blinding, of which only one trial set sham acupuncture in the control group and seven trials clarified the blinding of outcome assessors. Of the 13 studies reporting dropouts, 11 studies were rated as “high risk” because they did not apply a proper intention-to-treat analysis. All but one of these RCTs were not registered in advance and accessible for their protocols, so the reporting bias was rated as “unclear risk” for these studies. No other bias was detected for the included studies. A summary of the ROB assessment is shown in Figure 2. Meanwhile, there were plenty of items in the CONSORT (17/25 items) and the STRICTA (8/17 items) statement that did not achieve a desirable reporting rate (>80%), which is in accordance with the results from ROB measurements. The reporting quality of these RCTs can be found in Supplementary Tables 2, 3.

### Outcomes of Acupuncture for VCI

#### Global Cognitive Function

##### Mini-Mental State Examination

**Figure 3:**
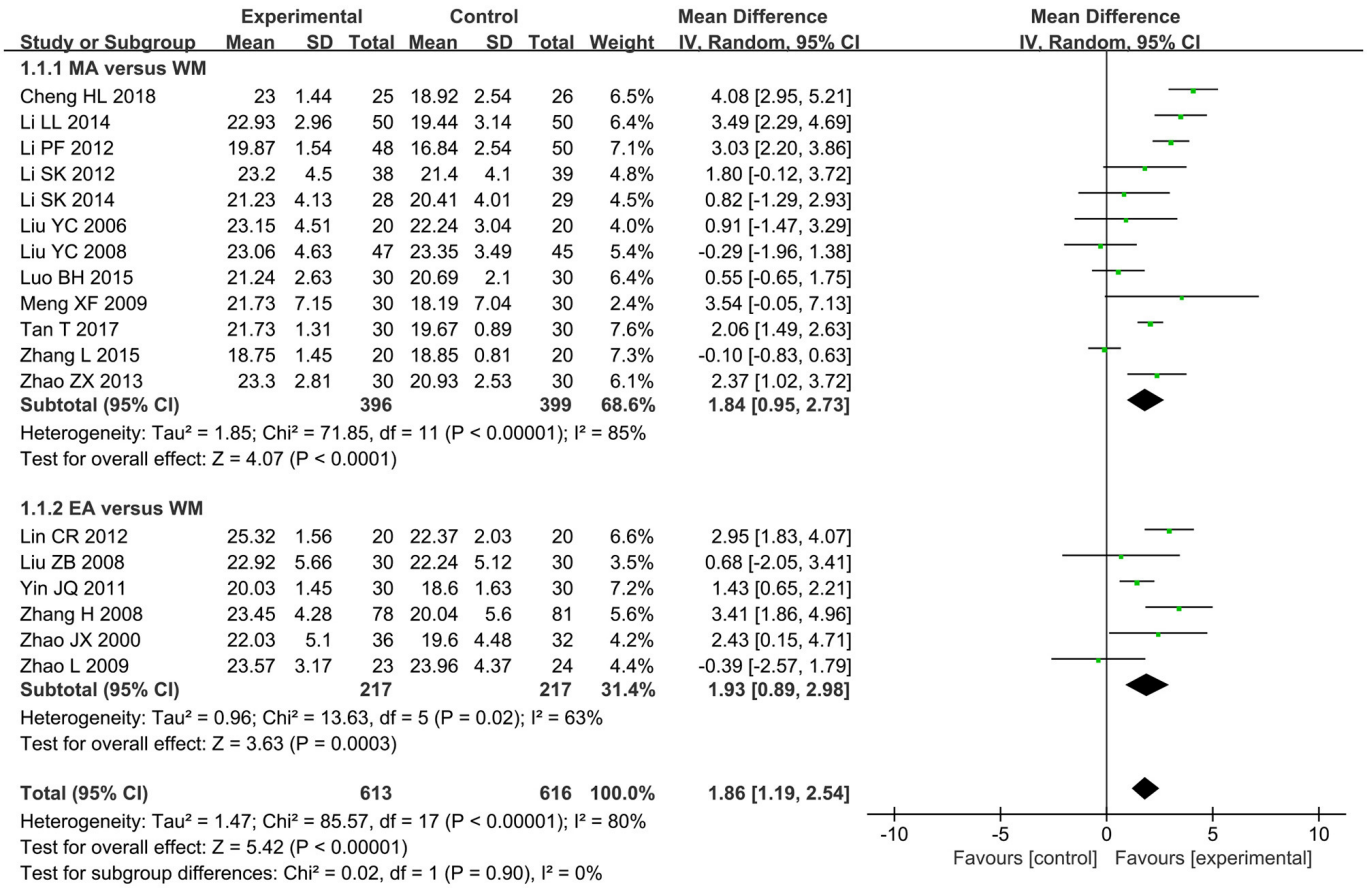
The forest plot of mini mental state examination (MMSE) comparing acupuncture vs. western medicine (WM).

Mini-mental state examination was the most frequently used scale to assess the effect of acupuncture on the global cognitive function for VCI. Information about MMSE comparing acupuncture with WM following treatment was available in 18 RCTs (Zhao et al., 2000, 2009; Liu et al., 2006; Liu Y. et al., 2008; Liu Z. B. et al., 2008; Zhang et al., 2008, 2015; Meng et al., 2009; Yin et al., 2011; Lin et al., 2012; Li P. et al., 2012; Li S. et al., 2012; Zhao and Xu, 2013; Li and Jiao, 2014; Li et al., 2014; Luo et al., 2015; Tan et al., 2017; Cheng et al., 2018). The pooled MD applying a RE model revealed a higher MMSE score for patients treated with acupuncture (MD 1.86, 95% CI 1.19–2.54, *I*^2^ = 80%, *p* < 0.00001, see Figure 3). With regard to the high heterogeneity shown in the result, a subgroup analysis was done based on different types of acupuncture, which could be divided into MA and EA. The subgroup analysis indicated that intra-subgroup heterogeneity remained high in both the subgroups (*I*^2^ = 85 and 63%), but the inter-subgroup heterogeneity was not too evident (*I*^2^ = 0%). The estimated MDs were 1.84 (95% CI 0.95–2.73, *p* < 0.0001, 12 studies) (Liu et al., 2006; Liu Y. et al., 2008; Meng et al., 2009; Li P. et al., 2012; Li S. et al., 2012; Zhao and Xu, 2013; Li and Jiao, 2014; Li et al., 2014; Luo et al., 2015; Zhang et al., 2015; Tan et al., 2017; Cheng et al., 2018) and 1.93 (95% CI 0.89–2.98, *p* = 0.0003, six studies) (Zhao et al., 2000, 2009; Liu Z. B. et al., 2008; Zhang et al., 2008; Yin et al., 2011; Lin et al., 2012) for the MA subgroup and the EA subgroup, respectively. Then, the sensitivity analysis was performed by removing the most weighted study. Meanwhile, a study was excluded at a time while the remaining RCTs were synthesized to identify which one yielded the heterogeneity and to evaluate whether the result would be changed by the omission of a single study. The results of sensitivity analyses confirmed that there was little impact on the pooled MD value but failed to figure out the heterogeneity maker because the level of heterogeneity was not reduced by omitting any studies. The inconsistent acupoint selection, treatment frequencies, and therapeutic duration among different studies might be responsible for the heterogeneity.

**Figure 4:**
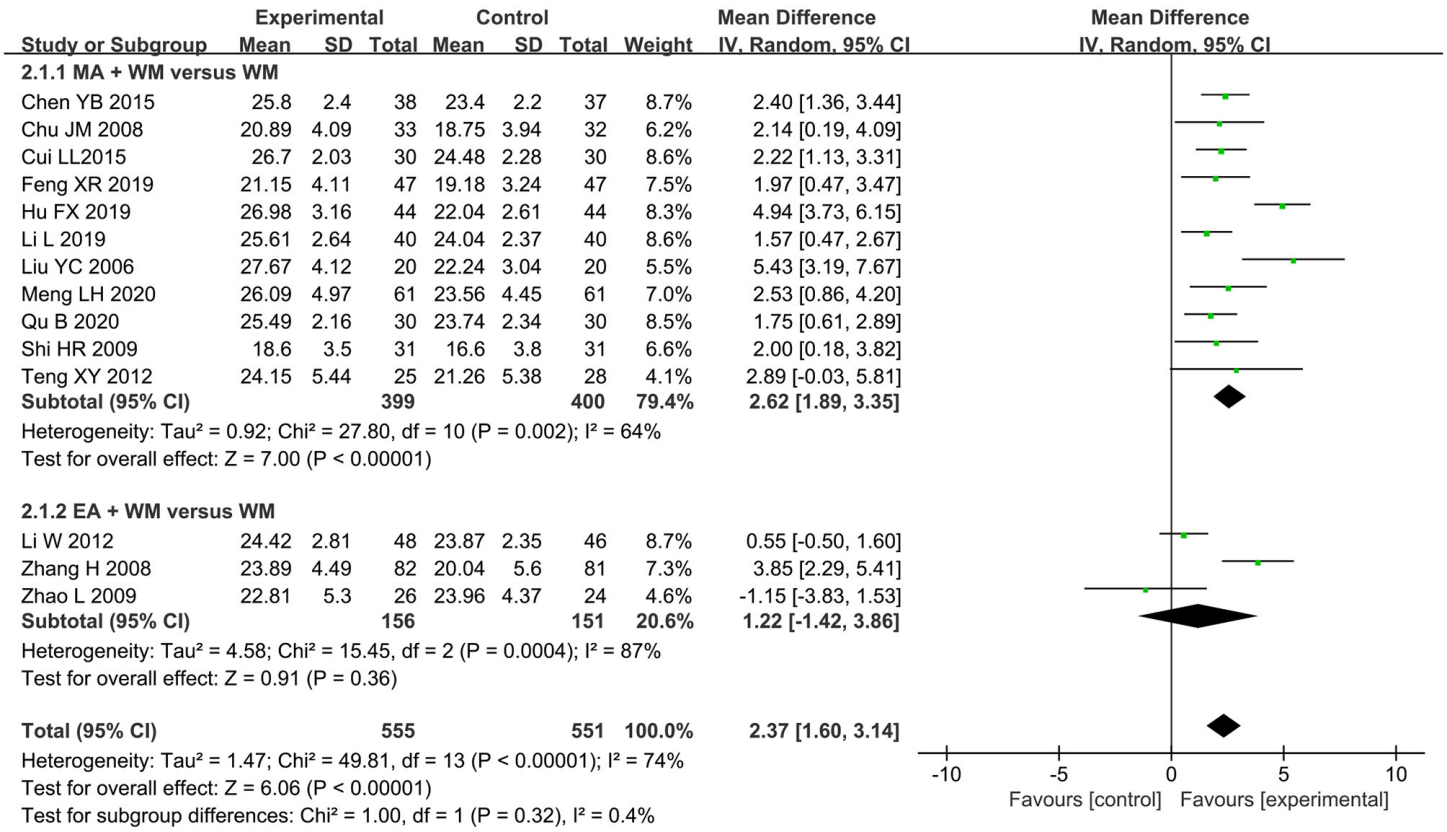
The forest plot of MMSE comparing acupuncture plus WM vs. WM alone.

Data from the 14 RCTs (Liu et al., 2006; Chu et al., 2008; Zhang et al., 2008; Shi et al., 2009; Zhao et al., 2009; Li W. et al., 2012; Teng and Lai, 2012; Chen et al., 2015; Cui et al., 2015; Feng et al., 2019; Hu et al., 2019; Li et al., 2019; Meng et al., 2020; Qu et al., 2020) displaying the higher scores in the MMSE scale were associated with a combination of acupuncture and WM when compared with WM alone with considerable heterogeneity (MD 2.37, 95% CI 1.6–3.14, *I*^2^ = 74%, *p* < 0.00001, see Figure 4). In acupuncture-type subgroup analyses, the pooled result favored MA plus WM with reduced heterogeneity (MD 2.62, 95% CI 1.89–3.35, *I*^2^ = 64%, *p* < 0.00001, 11 studies) (Liu et al., 2006; Chu et al., 2008; Shi et al., 2009; Teng and Lai, 2012; Chen et al., 2015; Cui et al., 2015; Feng et al., 2019; Hu et al., 2019; Li et al., 2019; Meng et al., 2020; Qu et al., 2020), whereas EA plus WM did not show better performance than WM alone with substantial heterogeneity (MD 1.22, 95% CI −1.42–3.86, *I*^2^ = 87%, *p* = 0.36, 3 studies) (Zhang et al., 2008; Zhao et al., 2009; Li W. et al., 2012). There was no significant subgroup effect between the two types of acupuncture (*p* = 0.32 and *I*^2^ = 0.4%). In sensitivity analyses, two trials were detected as the possible sources of heterogeneity in the MA plus WM subgroup and EA plus WM subgroup. Both subtotal and overall effect sizes were still steady with reduced heterogeneity after the corresponding studies were removed (see Supplementary Table 4). When compared with other studies, it led to a speculation that applying cluster needling at scalp acupoints in the trail of Hu FX (Hu et al., 2019) and the variant parameters of EA apparatus from others in the trail of Zhang H (Zhang et al., 2008) probably accounted for heterogeneity.

**Figure 5:**
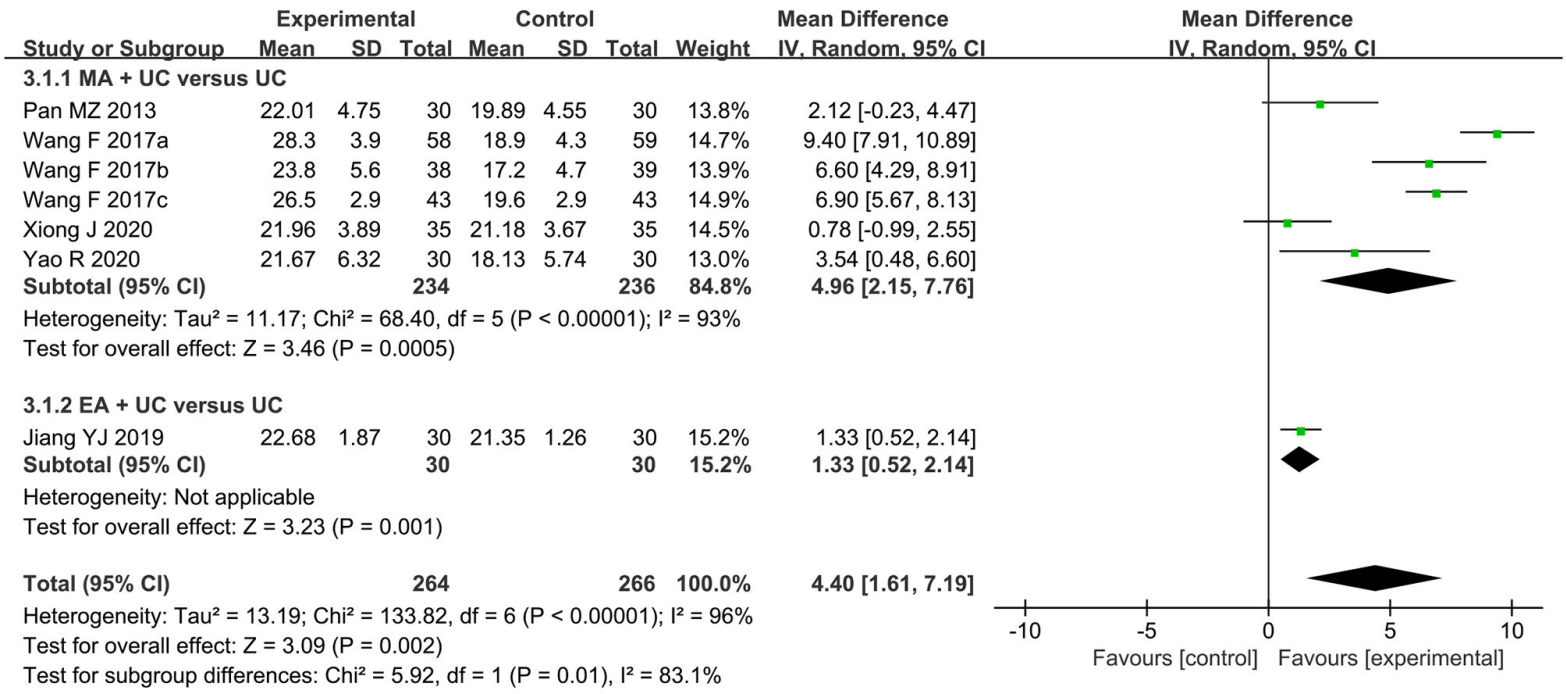
The forest plot of MMSE comparing acupuncture plus usual care (UC) vs. UC alone.

The meta-analysis result of seven RCTs (Pan and Ai, 2013; Wang and Wang, 2017a,b,c; Jiang et al., 2019; Xiong et al., 2020; Yao R. et al., 2020) demonstrated that the treatment with acupuncture plus UC was more beneficial in improving MMSE scores than UC alone (MD 4.4, 95% CI 1.61–7.19, *I*^2^ = 96%, *p* = 0.002, see Figure 5) with considerable intra- and inter-subgroup heterogeneity (*I*^2^ = 93 and 83.1%). The subgroup MDs were 4.96 (95% CI 2.15–7.76, *p* = 0.0005, six studies) (Pan and Ai, 2013; Wang and Wang, 2017a,b,c; Xiong et al., 2020; Yao R. et al., 2020) and 1.33 (95% CI 0.52–2.14, *p* = 0.001, one study) (Jiang et al., 2019) for the similar subgroups as mentioned above. Even though the heterogeneity was evident, sensitivity analyses could not decrease the level of heterogeneity in spite of the removal of any studies but could not unsettle the pooled result either. Unfortunately, the exact reasons for heterogeneity could not be determined because most of the included studies did not depict the details of UC or other conventional treatments adequately. The heterogeneity might be explained in part by that some trials arranged cognitive training as UC (Jiang et al., 2019; Xiong et al., 2020; Yao R. et al., 2020) while the others only used routine oral drugs for internal diseases.

#### Hasegawa's Dementia Scale

The Hasegawa's Dementia Scale was selected to evaluate the effect difference of acupuncture on a global cognitive function compared with WM in 12 RCTs (Mo et al., 2000a,b; Jiang et al., 2003; Liu et al., 2006; Niu and Liu, 2007; Liu Y. et al., 2008; Liu Z. B. et al., 2008; Chen et al., 2009; Li et al., 2009; Meng et al., 2009; Li P. et al., 2012; Cao et al., 2014). The pooled meta-analysis of the data showed a statistically significant difference between the experimental group and the control group (MD 2.2, 95% CI 1.27–3.13, *I*^2^ = 72%, *p* < 0.00001, see Supplementary Figure 1A). Subgroup analyses indicated that MDs were 1.83 (95% CI 0.79–2.87, *p* = 0.0006, *I*^2^ = 74%, eight studies) (Liu et al., 2006; Niu and Liu, 2007; Liu Y. et al., 2008; Chen et al., 2009; Li et al., 2009; Meng et al., 2009; Li P. et al., 2012; Cao et al., 2014) and 3.47 (95% CI 0.92–6.02, *p* = 0.008, *I*^2^ = 74%, four studies) (Mo et al., 2000a,b; Jiang et al., 2003; Liu Z. B. et al., 2008) for the MA and EA subgroup, respectively. The subgroup test suggested that there was no significant subgroup effect (*p* = 0.24, *I*^2^ = 26.4%) and demonstrated that the association between acupuncture and HDS improvement was independent of different acupuncture types. One trial (Li et al., 2009) contributed to the generation of heterogeneity in the MA subgroup. It has an unreported disease duration and a longer therapeutic course compared with other studies, which might be the reasons for heterogeneity. After this study was excluded, the pooled MD was still in favor of MA (see Supplementary Table 4). In the EA subgroup, the heterogeneity was not significantly reduced regardless of the removal of any study, but the pooled results showed no apparent fluctuations either.

The meta-analysis of four RCTs (Huang et al., 1992; Liu et al., 2006; Chu et al., 2008; Li W. et al., 2012) comparing acupuncture plus WM with WM alone indicated a higher HDS score in the experimental groups without apparent heterogeneity (MD 1.77, 95% CI 0.85–2.69, *I*^2^ = 31%, *p* = 0.0002, see Supplementary Figure 1B). There was also a statistically significant difference in the pooled MD of HDS scores when comparing acupuncture plus UC with UC alone (MD 6.42, 95% CI 5.04–7.81, *I*^2^ = 39%, *p* = 0.0002, two studies, see Supplementary Figure 1C) (Pan and Ai, 2013; Wang and Wang, 2017a). Meanwhile, the sensitivity analysis still validated the stability of the estimated results by omitting the maximum weighted studies.

#### Montreal Cognitive Assessment

Only one study (Zhang et al., 2019) reported that patients who received acupuncture treatment had a preferable effect in improving the MoCA score than those who received drug therapy (MD 3.16, 95% CI 1.5–4.82, *p* = 0.0002, see Supplementary Figure 2A). Using an RE model to pool together the results, four studies (Li et al., 2019; Chen et al., 2020; Meng et al., 2020; Qu et al., 2020) demonstrated that acupuncture combined with WM was more beneficial for patients with VCI than those with WM alone (MD 2.33, 95% CI 1.42–3.25, *I*^2^ = 68%, *p* < 0.00001, see Supplementary Figure 2B). A trial (Qu et al., 2020) was screened out by sensitivity analysis that exacerbated heterogeneity. The heterogeneity degree was decreased by omitting this study, which did not sacrifice the robustness of the final result (see Supplementary Table 4). Acupuncture plus UC also showed a better performance for the MoCA score compared with UC alone (MD 3.43, 95% CI 1.79–5.08, *I*^2^ = 0%, *p* < 0.0001, two studies, see Supplementary Figure 2C) (Jiang et al., 2019; Yao R. et al., 2020).

#### Alzheimer's Disease Assessment Scale-Cognitive Subscale

Two trials (Li and Jiao, 2014; Yang et al., 2019) focused on the therapeutic effect of acupuncture compared with WM; the pooled result indicated that patients with VCI who received acupuncture could attain more favorable improvements in the global cognitive function measured by the ADAS-cog scale (SMD −0.79, 95% CI −1.4 to −0.18, *I*^2^ = 83%, *p* = 0.01, see Supplementary Figure 3A). The heterogeneity between these two studies was caused by different degrees of VCI severity in the recruited patients and the distinct ADAS-cog scores at baseline, in which one trial included patients with mild to moderate vascular dementia (Li and Jiao, 2014), whereas the other trial collected cases with mild VCI and no dementia (Yang et al., 2019). As both studies reported positive conclusions, the sensitivity analysis did not alter the results regardless of the removal of any one of them (see Supplementary Table 4). However, another study (Yang et al., 2016) found that no significant difference existed between acupuncture plus WM and WM alone (MD −3.08, 95% CI −6.45–0.29, *p* = 0.07, see Supplementary Figure 3B).

#### General Skill Level on ADL

##### Activities of Daily Living Scale

**Figure 6:**
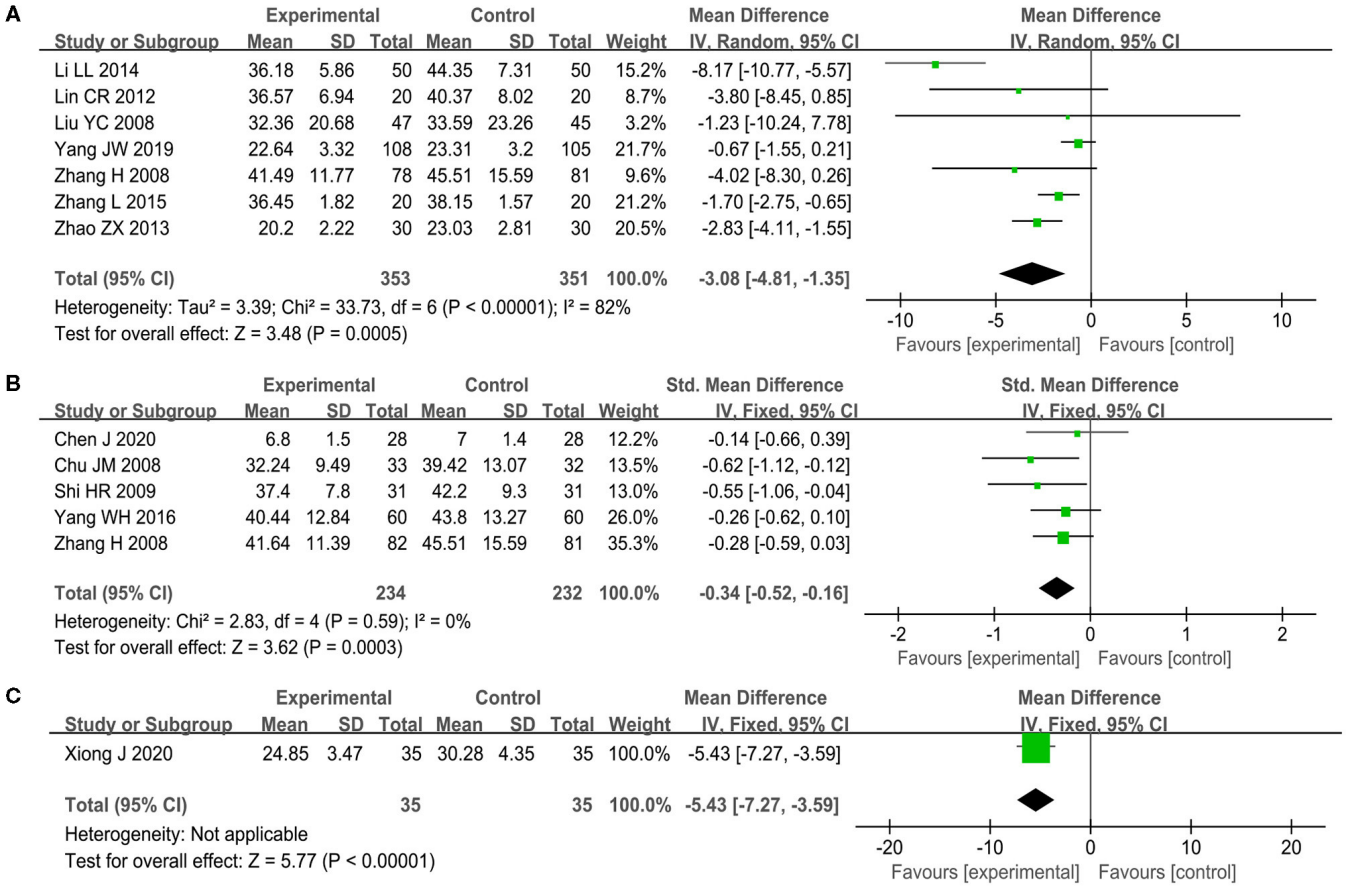
The forest plot of Activities of Daily Living (ADL) Scale. **(A)** Acupuncture vs. WM, **(B)** acupuncture plus WM vs. WM alone, and **(C)** acupuncture plus UC vs. UC alone.

12 RCTs (Chu et al., 2008; Liu Y. et al., 2008; Zhang et al., 2008, 2015; Shi et al., 2009; Lin et al., 2012; Zhao and Xu, 2013; Li and Jiao, 2014; Yang et al., 2016, 2019; Chen et al., 2020; Xiong et al., 2020) with 13 comparison pairs concentrated on the effect for the ADL Scale, which is comprised of the physical self-maintenance scale and the instrumental ADL Scale. A significant decrease on the ADL Scale was observed within the acupuncture group compared to the WM group by an RE meta-analysis (MD −3.08, 95% CI −4.81 to −1.35, *I*^2^ = 82%, *p* = 0.0005, seven studies, see Figure 6A) (Liu Y. et al., 2008; Zhang et al., 2008, 2015; Lin et al., 2012; Zhao and Xu, 2013; Li and Jiao, 2014; Yang et al., 2019). One trial (Li and Jiao, 2014) might be the likely source of heterogeneity, which did not change the sensitivity analysis result when it was excluded from the pooled process (see Supplementary Table 4). When comparing acupuncture plus WM with WM alone, a statistically significant difference was also revealed without an apparent degree of heterogeneity (SMD −0.34, 95% CI −0.52 to −0.16, *I*^2^ = 0%, *p* = 0.0003, five studies, see Figure 6B) (Chu et al., 2008; Zhang et al., 2008; Shi et al., 2009; Yang et al., 2016; Chen et al., 2020). One study (Xiong et al., 2020) showed that acupuncture plus UC got a better ADL Scale score than the UC alone group (MD −5.43, 95% CI −7.27 to −3.59, *p* < 0.0001, see Figure 6C).

#### Barthel ADL Index

Sufficient information was available in eight trials (Mo et al., 2000a,b; Jiang et al., 2003; Liu et al., 2006; Li P. et al., 2012; Li S. et al., 2012; Li et al., 2014; Zhang et al., 2019) which was estimated and pooled using a RE model showed that patients receiving acupuncture achieved better performance on BI than those who took drugs (MD 5.76, 95% CI 2.14–9.38, *I*^2^ = 60%, *p* = 0.002, see Supplementary Figure 4A). The sensitivity analysis indicated that one study (Li P. et al., 2012) accounted for major heterogeneity. After this study was removed, there was a reduction in heterogeneity while the robustness of the synthesized result was not changed (see Supplementary Table 4). The similar tendency of MD in BI was also demonstrated in the comparison between acupuncture plus WM and WM alone (MD 6.54, 95% CI 2.88–10.21, *I*^2^ = 79%, *p* = 0.002, seven studies, see Supplementary Figure 4B) (Liu et al., 2006; Li W. et al., 2012; Teng and Lai, 2012; Hu et al., 2019; Li et al., 2019; Meng et al., 2020; Qu et al., 2020) and between acupuncture plus UC and UC alone (MD 18.65, 95% CI 12.54–24.76, *I*^2^ = 95%, *p* < 0.00001, four studies, see Supplementary Figure 4C) (Wang and Wang, 2017a,b,c; Jiang et al., 2019). The sensitivity analysis pointed out the corresponding heterogeneity makers (Hu et al., 2019; Jiang et al., 2019) and confirmed the stability of the pooled results when these studies were singled out (see Supplementary Table 4). The variance of outcome measurement time points might be responsible for the substantial heterogeneity among the studies.

#### Functional Activities Questionnaire

Three RCTs (Mo et al., 2000a,b; Liu Z. B. et al., 2008) included information regarding the FAQ, whose pooled result using a FE model indicated that no significant effect difference was shown between acupuncture and WM (MD −0.84, 95% CI −2.41–0.74, *I*^2^ = 0%, *p* = 0.3, see Supplementary Figure 5).

#### Safety and AEs

**Figure 7:**
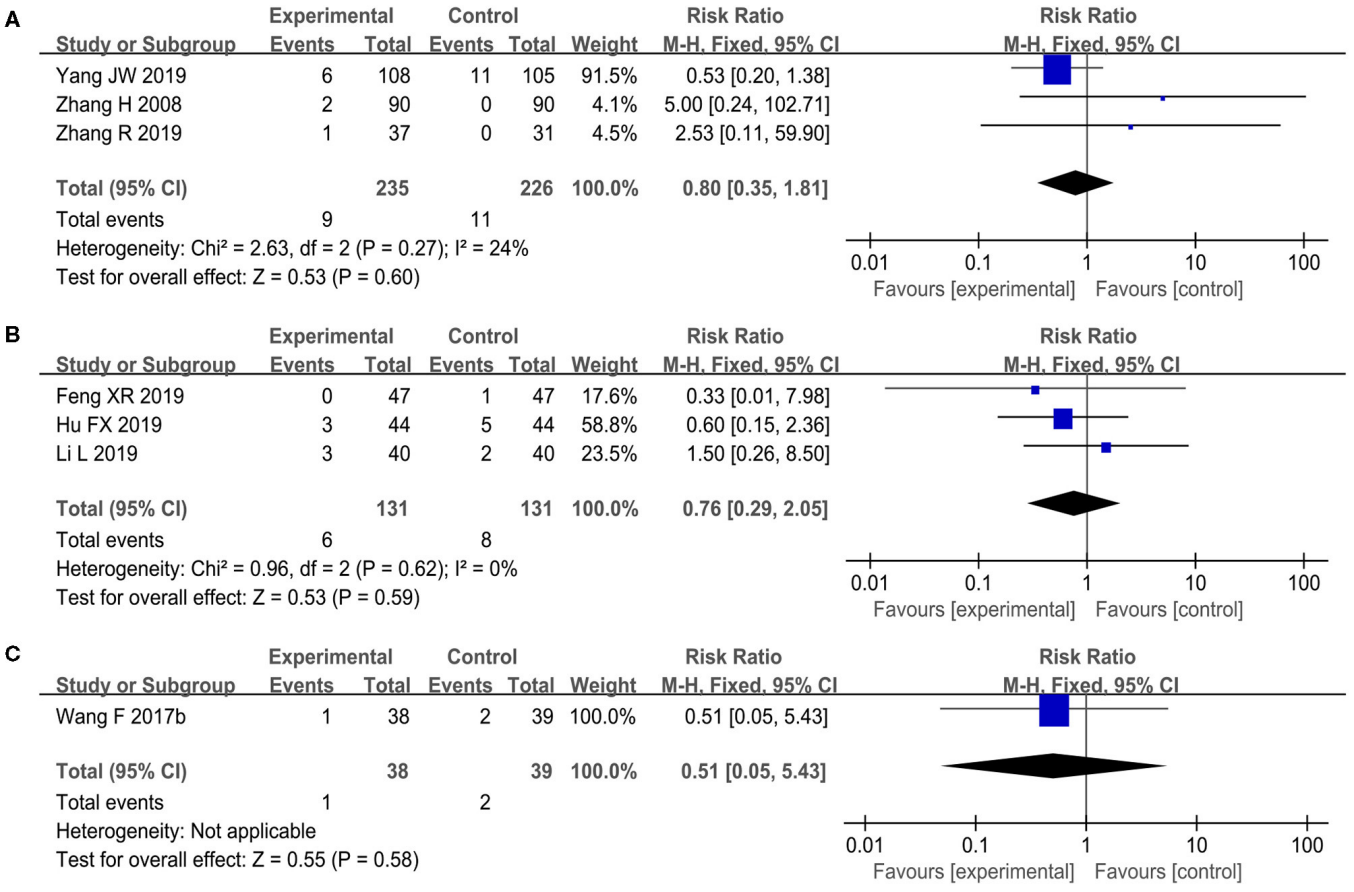
The forest plot of incidence of adverse events (AEs). **(A)** Acupuncture vs. WM, **(B)** acupuncture plus WM vs. WM alone, and **(C)** acupuncture plus UC vs. UC alone.

Safety assessments were mentioned in 10 studies (Liu Y. et al., 2008; Zhang et al., 2008, 2019; Li W. et al., 2012; Tan et al., 2017; Wang and Wang, 2017b; Feng et al., 2019; Hu et al., 2019; Li et al., 2019; Yang et al., 2019), of which 7 studies explicitly described the specific AEs and exact number of episodes (Zhang et al., 2008, 2019; Wang and Wang, 2017b; Feng et al., 2019; Hu et al., 2019; Li et al., 2019; Yang et al., 2019), while the other 3 studies reported no obvious treatment-related side effects (Liu Y. et al., 2008; Li W. et al., 2012; Tan et al., 2017). Under acceptable heterogeneity, three RCTs (Zhang et al., 2008, 2019; Yang et al., 2019) comparing acupuncture with WM found no statistically significant difference in the occurrence of AEs (RR 0.8, 95% CI 0.35–1.81, *I*^2^ = 24%, *p* = 0.6, see Figure 7A) as well as three other RCTs (Feng et al., 2019; Hu et al., 2019; Li et al., 2019) comparing acupuncture plus WM with WM alone (RR 0.76, 95% CI 0.29–2.05, *I*^2^ = 0%, *p* = 0.59, see Figure 7B). One trial (Wang and Wang, 2017b) reported that acupuncture plus UC made no difference in the incidence of AEs compared with UC alone (RR 0.51, 95% CI 0.05–5.43, *p* = 0.58, see Figure 7C). In the 37/800 cases, there were four kinds of AEs including fainting during acupuncture (*n* = 9), pain or bruising at the sites of needle insertion (*n* = 6), nausea or vomiting caused in the control groups (*n* = 21), and bone fracture (*n* = 1). Most of the AEs were mild and the participants could recover spontaneously without the need for specific medical evaluation and intervention, except for the case of bone fracture due to tumbling in the acupuncture group (Yang et al., 2019).

### Publication Bias and Quality of Evidence

**Figure 8:**
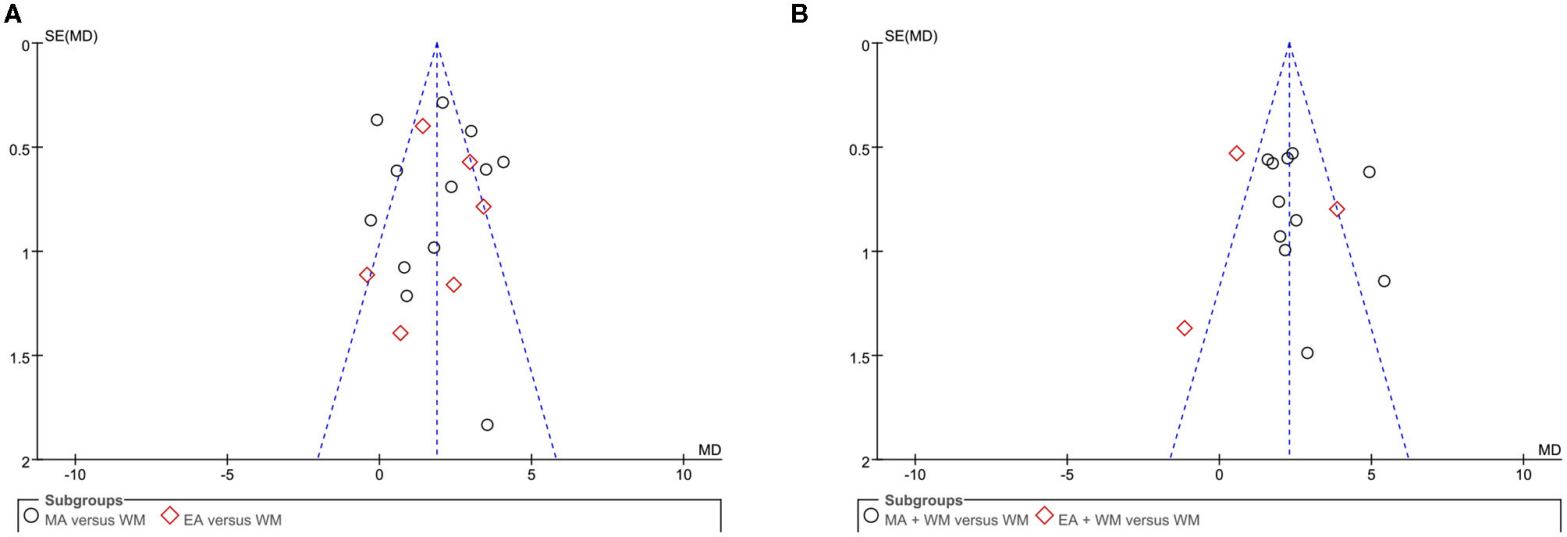
The funnel plot of MMSE. **(A)** Acupuncture vs. WM and **(B)** acupuncture plus WM vs. WM alone.

There were two comparisons between acupuncture vs. WM in the meta-analysis of MMSE and HDS, as well as another comparison of acupuncture plus WM vs. WM alone for MMSE that included more than 10 studies. Publication bias assessments are presented as funnel plots (see Figure 8 and Supplementary Figure 6). From the roughly symmetrical shapes of these funnel plots, no obvious publication bias was observed. The overall strength of evidence acquired from the included RCTs was indicated with a “low” or “very low” level of certainty that acupuncture was associated with improved mental state and cognition for patients with VCI measured using MMSE and HDS, respectively (see Table 3).

**Table 3:** Summary of findings and strength of evidence for outcomes.

**Patient or population**: Patients with vascular cognitive impairment (VCI) **Setting**: Hospitals in mainland China				
**Outcomes**	Anticipated absolute effects^*^ **(95% CI)**		***N*** **o̱ of participants (studies)**	**Certainty of the evidence (GRADE)**
	**Risk with Western medicine**	**Risk with acupuncture**		
**Acupuncture compared to Western medicine for VCI** **Intervention**: Acupuncture **Comparison**: Western medicine				
Mental state (MMSE) assessed with: MMSE scale (higher score better outcome) Scale from: 0 to 30 follow up: 2–12 weeks	The mean mental state ranged from **16.84** to **23.96**	MD **1.86 higher** (1.19 higher to 2.54 higher)	1,229 (18 RCTs)	⊕⊕○○ LOW^a^^,^^b^
Cognition (HDS) assessed with: HDS Chinese version (higher score better outcome) Scale from: 0 to 30 follow up: 4–10 weeks	The mean cognition ranged from **12.68** to **24.36**	MD **2.2 higher** (1.27 higher to 3.13 higher)	781 (12 RCTs)	⊕⊕○○ LOW^a^^,^^b^
**Acupuncture plus Western medicine compared to Western medicine for VCI** **Intervention**: Acupuncture plus Western medicine **Comparison**: Western medicine				
Mental state (MMSE) assessed with: MMSE scale (higher score better outcome) Scale from: 0 to 30 follow up: 4–12 weeks	The mean mental state ranged from **16.6** to **24.48**	MD **2.37 higher** (1.6 higher to 3.14 higher)	1,106 (14 RCTs)	⊕⊕○○ LOW^a^^,^^b^
Cognition (HDS) assessed with: HDS Chinese version (higher score better outcome) Scale from: 0 to 30 follow up: 2–12 weeks	The mean cognition ranged from **17.12** to **26.83**	MD **1.77 higher** (0.85 higher to 2.69 higher)	235 (4 RCTs)	⊕○○○ VERY LOW^a^^,^^c^^,^^b^
**Acupuncture plus Usual care compared to Usual care for VCI** **Intervention**: Acupuncture plus Usual care **Comparison**: Usual care				
Mental state (MMSE) assessed with: MMSE scale (higher score better outcome) Scale from: 0 to 30 follow up: 4–12 weeks	The mean mental state ranged from **17.2** to **21.35**	MD **4.4 higher** (1.61 higher to 7.19 higher)	530 (7 RCTs)	⊕○○○ VERY LOW^a^^,^^b^^,^^d^
Cognition (HDS) assessed with: HDS Chinese version (higher score better outcome) Scale from: 0 to 30 follow up: 4–10 weeks	The mean cognition ranged from **18.6** to **19.8**	MD **6.42 higher** (5.04 higher to 7.81 higher)	177 (2 RCTs)	⊕○○○ VERY LOW^a^^,^^c^^,^^b^
GRADE working froup grades of evidence High certainty: We are very confident that the true effect lies close to that of the estimate of the effect. Moderate certainty: We are moderately confident in the effect estimate: The true effect is likely to be close to the estimate of the effect, but there is a possibility that it is substantially different. Low certainty: Our confidence in the effect estimate is limited: The true effect may be substantially different from the estimate of the effect. Very low certainty: We have very little confidence in the effect estimate: The true effect is likely to be substantially different from the estimate of effect.				

* *The risk in the intervention group (and its 95% CI) is based on the assumed risk in the comparison group and the **relative effect** of the intervention (and its 95% CI)*.

*CI, Confidence interval; HDS, Hasegawa's Dementia Scale; MD, Mean difference; MMSE, Mini Mental State Examination*.

a *Most of the RCTs were low quality with an inadequate level of blinding and unclear risk of concealment of allocation*.

b *The statistical test for heterogeneity showed that large variation (I^2^ > 50%) existed in point estimates due to among-study differences*.

c *The total sample size was lower than 400*.

d *Study protocols were inaccessible and the number of RCTs was insufficient for a publication bias estimation*.

## Discussion

### Summary of Main Findings

In this SR, 48 RCTs involving 3,778 patients in total were included for meta-analysis. The merged data indicated that acupuncture is more beneficial for improving the global cognitive function (measured by MMSE, HDS, MoCA, and ADAS-cog) and daily performance (measured by ADL Scale and BI) of patients with VCI compared with WM. Favorable results were also observed when acupuncture was combined with WM or UC in comparison with the matching control conditions. Furthermore, the subgroup analysis did not show an effect difference on the cognition (measured by MMSE and HDS) between MA and EA when comparing acupuncture with WM. There were no significant differences in the incidence of AEs between the acupuncture group and the control group. Even though the certainty of the evidence was low or very low due to the generally poor methodological quality and substantial heterogeneity among studies, this present review provides an updated synthesis of the existing RCT results of acupuncture for VCI and points out the remaining research gaps that are necessary to be filled.

### Applicability for Clinical Practice

This SR and meta-analysis revealed that applying acupuncture might serve as a monotherapy or an adjuvant therapy to improve general cognitive functions and ADL of patients with VCI as well their safety. Different from AD, up to now, there is still no available symptomatic pharmacological therapy for VCI (including vascular dementia) licensed by the authoritative official administration (National Collaborating Centre for Mental Health, 2018; van der Flier et al., 2018). As the values brought by pharmacological therapies have demonstrated very limited and possible side effects, which sometimes counterbalance their benefits (Wilkinson et al., 2003; Kavirajan and Schneider, 2007), the demand for seeking evidence-based alternative interventions has transitioned gradually from being necessary to urgent. Additionally, many medical societies show a rising interest in how non-pharmacological interventions could help patients with dementia to maintain daily functional independence (Yao S. et al., 2020). Physical approaches such as acupuncture seem to be desirable for patients with cognitive disorders, especially for those who ask for long-term treatment (Su et al., 2020a). According to the clinical evidence, acupuncture is a relatively resource-intensive intervention, which is particularly suitable for chronic diseases (Lothgren and Zethraeus, 2000; Raftery, 2001; Wonderling et al., 2004). Therefore, as a relatively cost-effective clinical therapy, which could ease the economic and medical burden, acupuncture warrants further investigation for its potential benefits. For patients with VCI and healthcare workers, our results may provide some new insights into clinical practice. Unfortunately, based on the RCTs with the generally problematic methodology, which shook the reliability of findings, the review could temporally provide weak evidence to support the routine application of acupuncture as a monotherapy or an adjunctive therapy to WM or UC, or both, in treating patients with VCI.

From a clinical perspective, assessing the effects of a therapy for VCI must consist of not only cognitive or mental outcomes but also the quality of physical and social life (Gorelick et al., 2011). Accompanied by the deterioration of cognition, ADL of patients would be unavoidably and significantly affected. How to integrate symptomatic management with ADL improvement for patients remains a challenge (Desai et al., 2004). The pooled results indicated that the clinical effects of acupuncture for VCI might be multi-dimensional, which means that acupuncture is not a standalone approach for cognitive improvement. Most of the included RCTs evaluated the effects of acupuncture on ADL according to different scales (ADL Scale or BI) and found that acupuncture did promote ADL recovery together with cognitive improvement at the same time. However, another common and knotty management issue accompanied by the lasting VCI is psychiatric disorders (for example, agitation, aggression, or psychosis) (Wang et al., 2005), which were controversial with the use of some antipsychotics but were not paid close attention to the published acupuncture papers. Hence, currently, our review cannot provide any conclusion regarding whether acupuncture may alleviate the symptoms in this aspect.

An in-depth descriptive analysis based on the included RCTs indicated the following acupuncture regimens for VCI despite the great diversity existing in the detailed protocols. The frequently applied local points were *Bai-hui* (GV20), *Si-shen-cong* (EX-HN1), *Shen-ting* (GV24), *Feng-chi* (GB20), *Shui-gou* (GV26), and *Ben-shen* (GB13), whereas the frequently chosen distant points were *Nei-guan* (PC6), *Zu-san-li* (ST36), *San-yin-jiao* (SP6), and *Tai-xi* (KI3). According to the meridian system of acupuncture based on TCM, the GV Meridian plays an essential role in modulating cerebral function including cognition (Su et al., 2020b). The primary acupoints selected are those who belong to DV Meridian and distribute on the craniofacial region, whereas the auxiliary acupoints are those essential acupoints that can lift spirit, clear mind, or promote resuscitation and distribute on the limbs. This pattern of acupoint selection and combination is highly consistent with the TCM theory. In addition, flexible formulas according to different phases, TCM syndromes, and personal characteristics, namely tailored diagnosis and treatment, were allied in 18 trials, which are kept in line with the recommendation from our expert consensus survey (Su et al., 2020a). Given that VCI is a progressive geriatric disease, most researchers preferred a longer retention time, more frequent sessions, and a longer therapeutic course to ensure adequate acupuncture stimulation. In addition, 30 min of needle retention time was recommended. As the main purpose of our SR was not to compare the effect difference under different acupuncture techniques and did not include these kind of studies, the acupuncture regimens summarized above can be just regarded as a reference for acupuncturists to implement their daily practice and for researchers to design an ideal acupuncture protocol in their new studies.

### Implication for Future RCTs

As acupuncture literatures are mainly published in Chinese journals, it is indispensable to search the Chinese databases for collecting the relevant RCTs as comprehensively as possible. However, it was frustrating to find that most of the included studies published in the Chinese language showed critically low quality in the methodological design and reporting completeness. On evaluation by the CONSORT statement and the STRICTA checklist, plenty of items did not reach a satisfactory reporting rate (>80%). The key RCT elements such as random sequence generation, allocation concealment, and blinding were not mentioned or correctly depicted in more than two-thirds of the literatures.

Adequately designed studies with rigorous methods are of necessity to objectively appraise the real effects of acupuncture for VCI and provide high-grade evidence for clinical practice at last. Thus, the CONSORT statement (Schulz et al., 2010), as a standard guideline to facilitate the RCT design and orchestrate a complete and transparent report, should be recommended for future conducted trials. Furthermore, given that only one trial in this review was preregistered (Yang et al., 2019), prospective registration of the research protocol in registry centers (such as Chinese Clinical Trials Registry, and ClinicalTrials) should be strongly and urgently appealed so that their studies can be tracked by others. Additionally, preregistration is also conducive to eliminate suspicions about reporting bias. With regard to blinding, even though blinding intervenors is almost impractical for acupuncture RCTs due to its inherent feature as a non-pharmaceutical therapy, it is of necessity and feasiblity to blind patients and outcome assessors in particular. Nonetheless, despite seven trials using UC as a comparator in this review, only one team applied sham acupuncture in the control group simultaneously for blinding (Xiong et al., 2020). Even if comparing acupuncture with WM, researchers can still take into consideration adding simulated placebos in the experimental group and sham acupuncture in the control group to blind patients with VCI. As for instruments or scales to assess the effectiveness for VCI, the options are multiple and open for investigators so long as they can give rationales (O'Brien and Thomas, 2015; Dichgans and Leys, 2017; van der Flier et al., 2018), but participants with cognitive impairment are sometimes poor reporters for their own symptoms, especially when they are suffering from moderate or severe dementia. Therefore, a third assessor should assume responsibility for the assessment, and blinding assessors to treatment allocation is essential to avoid detection bias (Chan et al., 2018).

Of note, there were apparent heterogeneities among the included studies, so the RE model was frequently applied in this review. Although we scheduled the acupuncture style as a stratified factor, heterogeneity could not be significantly reduced by the subgroup analysis between the MA group and the EA group. This is likely to be associated with an unbalance due to acupuncture-related parameters such as acupoint selection and combination, treatment frequency, needling retention, as well as the qualifications and technical skills of acupuncturists. Even if the studies chose the same acupuncture style, these parameters were still variable and had an impact on the holistic effectiveness of acupuncture. Without a clear and an elaborated description of their acupuncture protocols, we failed to draw reliable judgments on the causes of heterogeneity but just speculated the possible studies that might account for the higher heterogeneity by sensitivity analyses. Therefore, the STRICTA reporting checklist (MacPherson and Jobst, 2010), as an extension of the CONSORT statement specially designed for acupuncture interventions, including not only the crucial components of needling details but also the precise description required for control interventions, should be followed to further improve the reporting completeness. With the assistance of the STRICTA checklist, acupuncture RCTs can be more accurately interpreted and more easily replicated. As for future SRs, it may be also beneficial for better analyzing the reasons for unexplained heterogeneity.

### Findings in Relation to Previous SRs

A recently published SR concerning acupuncture for AD revealed that acupuncture did not achieve superior outcomes than drug treatment, which is contrary to our findings in this review (Wang et al., 2020). The discrepancy may be associated with the fact that the pharmacological strategy for AD places emphasis on compensating cholinergic deficiency by using cholinesterase inhibitors such as donepezil, rivastigmine, and galantamine, which have been universally recommended (Kaduszkiewicz et al., 2005), but whether these drugs can still work for VCI is debatable (Dichgans and Leys, 2017). Additionally, this review also found that acupuncture plus drug therapy might be more beneficial in improving the global cognitive function than drug therapy alone, which is consistent with our results.

There are two previously published SRs referring to acupuncture in treating VCI (Peng et al., 2007; You et al., 2017). However, due to no eligible trials in compliance with the stringent inclusion criteria of the authors, one Cochrane SR exploring the efficacy and safety of acupuncture for VCI did not include any RCTs and achieved no conclusive results (Peng et al., 2007). This SR has not been updated so far since 2011, even though, in the recent decade, new RCTs emerged in large numbers. Another SR published in 2017 was conducted to assess the RCT reporting quality of SA for VCI, but SA is just one style of acupuncture therapy and the therapeutic effects were not evaluated in this review (You et al., 2017). Meanwhile, other past reviews also focused on acupuncture for one subtype of vascular cognitive disorders, including post-stroke cognitive impairment (Liu et al., 2014; Zhou et al., 2020) or vascular mild cognitive impairment (Cao et al., 2013; Deng and Wang, 2016). Given that VCI is a kind of complicated disease involving different pathogenesis and phases, our study paid more attention to the overall effects of acupuncture for VCI and comprehensively assessed global cognition and ADL performance. Notwithstanding, our results were in accordance with the earlier SRs, which involved only one subtype or a certain degree of severity of patients with VCI.

### Limitations

There are several limitations in this SR that need to be considered. The primary limitation is that even though 48 RCTs were included eventually, the general methodological and reporting quality of these studies was poor and unsatisfactory. Many authors did not specify the details of their randomization procedures, allocation concealment, and assessor blinding, which resulted in a certain degree of potential selection bias and detection bias. Moreover, only one trial established sham acupuncture in the control group aiming to blind the participants. Given that most of the outcomes were calculated from the participant-reported information, it inevitably produced a high risk of performance bias. Additionally, the fact that only one trial was formally registered induced the dubiety of reporting bias as well. The lack of high-quality RCTs unavoidably hindered the reliable evaluation of the effectiveness of acupuncture. The second limitation was the observation of considerable heterogeneity. Even though subgroup analyses were attempted to reduce the heterogeneity for different acupuncture styles, sensitivity analyses were applied to screen out the likely heterogeneity maker, acupuncture, as a complicated intervention, and the success of acupuncture in treatment depends on multiple elements, which always differed among studies. As VCI is a disease with highly intricate characteristics as well, the pathogenesis, phase of care, and comorbidity are the possible factors in the variability of estimations. Some studies within specific areas are suggested to thoroughly judge how and to what extent heterogeneity is ascribed to these factors. Both limitations led to the ultimate grade downloading of the evidence in this review, which makes the results to be interpreted with caution. A further limitation is that all RCTs were conducted in China and that the acupuncture therapy was mainly based on the TCM theory, although of which six articles were published in English. Up to now, there are many forms or styles of acupuncture. While TCM style acupuncture is probably the most commonly used style of acupuncture in many countries, it uses diagnoses and treatment techniques that are quite different from other traditional acupuncture styles that are practiced, for example, in Japan, Korea, Australia, the UK, the USA, Europe, etc. (Su et al., 2021). The databases used in this SR are limited to only English or Chinese publication. It should be recognized that a few literatures on acupuncture can be published in other languages as well. Therefore, this may implicate additional publication bias and inhibit the results from extrapolating to other extensive regions. Therefore, more trials are expected in the future to explore the real applicability of acupuncture.

## Conclusions

Current evidence is insufficient for effective VCI management. This review suggests that acupuncture as a monotherapy or an adjuvant therapy may play a positive role in improving the cognition and daily performance of patients with VCI associated with few side effects. Different styles may not significantly influence its effectiveness. More rigorously designed and preregistered RCTs are highly desirable to verify the therapeutic benefits and determine an optimal acupuncture paradigm. The methodological and reporting quality of future researches should be enhanced by adhering to authoritative standardized statements.

## Data Availability Statement

The original contributions presented in the study are included in the article/Supplementary Material, further inquiries can be directed to the corresponding author/s.

## Author Contributions

X-TS and C-ZL put forward this review and designed the protocol. L-QW determined the inclusion eligibility of RCTs. X-TS and NS carried out the literature retrieval, study selection, and data extraction. NZ and XZ performed the ROB assessment. J-LL drew the figures and summarized the tables. X-TS wrote the initial manuscript. G-XS and J-WY further modified and polished the article. All authors have read this article and agreed with the presented findings.

## Conflict of Interest

The authors declare that the research was conducted in the absence of any commercial or financial relationships that could be construed as a potential conflict of interest. The reviewer LZ declared a shared affiliation, with no collaboration, with one of the authors NS to the handling editor at the time of the review.

## Publisher's Note

All claims expressed in this article are solely those of the authors and do not necessarily represent those of their affiliated organizations, or those of the publisher, the editors and the reviewers. Any product that may be evaluated in this article, or claim that may be made by its manufacturer, is not guaranteed or endorsed by the publisher.
