# Supplementary material for: Effectiveness and Safety of Acupuncture for Vascular Cognitive Impairment: A Systematic Review and Meta-Analysis
Source: Front Aging Neurosci. 2021 Aug 6;13:692508. doi: 10.3389/fnagi.2021.692508 (PMC8377366; doi:10.3389/fnagi.2021.692508)
Supplement: Supplementary file 1 [file Table_1.pdf]

Supplementary Material

Effectiveness and Safety of Acupuncture for Vascular Cognitive Impairment: A Systematic Review and Meta-Analysis

Xin-Tong Su[1,2], Ning Sun[3], Na Zhang[4], Li-Qiong Wang[1], Xuan Zou[1], Jin-Ling Li[1], Jing-Wen Yang[1], Guang-Xia Shi[1*] and Cun-Zhi Liu[1,2*]

[1] *International Acupuncture and Moxibustion Innovation Institute, School of Acupuncture-Moxibustion and Tuina, Beijing University of Chinese Medicine, Beijing, China*

[2] *Traditional Chinese Medicine (TCM) in the Prevention and Rehabilitation of Stroke Task Force, World Stroke Organization, Geneva, Switzerland*

[3] *Acupuncture and Tuina School/The 3rd Teaching Hospital, Chengdu University of Traditional Chinese Medicine, Chengdu, China*

[4] *School of Acupuncture-Moxibustion and Tuina, Shandong University of Chinese Medicine, Jinan, China*

Supplementary Table 1. Preferred Reporting Items for Systematic Reviews and Meta-Analyses (PRISMA) statement

**Section/topic**	**#**	**Checklist item**	**Reported on page #**
**TITLE**			
Title	1	Identify the report as a systematic review, meta-analysis, or both.	1
**ABSTRACT**			
Structured summary	2	Provide a structured summary including, as applicable: background; objectives; data sources; study eligibility criteria, participants, and interventions; study appraisal and synthesis methods; results; limitations; conclusions and implications of key findings; systematic review registration number.	1-2
**INTRODUCTION**			
Rationale	3	Describe the rationale for the review in the context of what is already known.	2
Objectives	4	Provide an explicit statement of questions being addressed with reference to participants, interventions, comparisons, outcomes, and study design (PICOS).	2
**METHODS**			
Protocol and registration	5	Indicate if a review protocol exists, if and where it can be accessed (e.g., Web address), and, if available, provide registration information including registration number.	3
Eligibility criteria	6	Specify study characteristics (e.g., PICOS, length of follow-up) and report characteristics (e.g., years considered, language, publication status) used as criteria for eligibility, giving rationale.	3
Information sources	7	Describe all information sources (e.g., databases with dates of coverage, contact with study authors to identify additional studies) in the search and date last searched.	3
Search	8	Present full electronic search strategy for at least one database, including any limits used, such that it could be repeated.	3
Study selection	9	State the process for selecting studies (i.e., screening, eligibility, included in systematic review, and, if applicable, included in the meta-analysis).	3
Data collection process	10	Describe method of data extraction from reports (e.g., piloted forms, independently, in duplicate) and any processes for obtaining and confirming data from investigators.	3
Data items	11	List and define all variables for which data were sought (e.g., PICOS, funding sources) and any assumptions and simplifications made.	3

Risk of bias in individual studies	12	Describe methods used for assessing risk of bias of individual studies (including specification of whether this was done at the study or outcome level), and how this information is to be used in any data synthesis.	3-4
Summary measures	13	State the principal summary measures (e.g., risk ratio, difference in means).	4
Synthesis of results	14	Describe the methods of handling data and combining results of studies, if done, including measures of consistency (e.g., I^2) for each meta-analysis.	4
Risk of bias across studies	15	Specify any assessment of risk of bias that may affect the cumulative evidence (e.g., publication bias, selective reporting within studies).	3-4
Additional analyses	16	Describe methods of additional analyses (e.g., sensitivity or subgroup analyses, meta-regression), if done, indicating which were pre-specified.	4
**RESULTS**			
Study selection	17	Give numbers of studies screened, assessed for eligibility, and included in the review, with reasons for exclusions at each stage, ideally with a flow diagram.	4-5
Study characteristics	18	For each study, present characteristics for which data were extracted (e.g., study size, PICOS, follow-up period) and provide the citations.	6-7
Risk of bias within studies	19	Present data on risk of bias of each study and, if available, any outcome level assessment (see item 12).	4-5, 10
Results of individual studies	20	For all outcomes considered (benefits or harms), present, for each study: (a) simple summary data for each intervention group (b) effect estimates and confidence intervals, ideally with a forest plot.	5, 9-14
Synthesis of results	21	Present results of each meta-analysis done, including confidence intervals and measures of consistency.	5, 9-14
Risk of bias across studies	22	Present results of any assessment of risk of bias across studies (see Item 15).	4-5, 10
Additional analysis	23	Give results of additional analyses, if done (e.g., sensitivity or subgroup analyses, meta-regression [see Item 16]).	5, 9-14
**DISCUSSION**			
Summary of evidence	24	Summarize the main findings including the strength of evidence for each main outcome; consider their relevance to key groups (e.g., healthcare providers, users, and policy makers).	14-17
Limitations	25	Discuss limitations at study and outcome level (e.g., risk of bias), and at review-level (e.g., incomplete retrieval of identified research, reporting bias).	17-18
Conclusions	26	Provide a general interpretation of the results in the context of other evidence, and implications for future research.	18
**FUNDING**			

Funding	27	Describe sources of funding for the systematic review and other support (e.g., supply of data); role of funders for the systematic review.	18

Supplementary Table 2. Rating of overall quality using items from the CONSORT guideline (n = 48)			
**Criteria**	**Description**	**Number of positive trials (n)**	**Percentage (%)**
**Title and abstract**	1a Identification as a randomised trial in the title	4	8.33
	1b Structured summary of trial design, methods, results, and conclusions	45	93.75
**Introduction**			
Background and objectives	2a Scientific background and explanation of rationale	29	60.42
	2b Specific objectives or hypotheses	20	41.67
**Methods**			
Trial design	3a Description of trial design (such as parallel, factorial) including allocation ratio	6	12.5
	3b Important changes to methods after trial commencement (such as eligibility criteria), with reasons	0	0
Participants	4a Eligibility criteria for participants	46	95.83
	4b Settings and locations where the data were collected	43	89.58
Interventions	5 The interventions for each group with sufficient details to allow replication, including how and when they were actually administered	48	100
Outcomes	6a Completely defined prespecified primary and secondary outcome measures, including how and when they were assessed	13	27.08
	6b Any changes to trial outcomes after the trial commenced, with reasons	0	0
Sample size	7a How sample size was determined	2	4.17
	7b When applicable, explanation of any interim analyses and stopping guidelines	0	0
Randomisation	8a Method used to generate the random allocation sequence	32	66.67
	8b Type of randomisation; details of any restriction (such as blocking and block size)	6	12.5
Allocation concealment	9 Mechanism used to implement the random allocation sequence (such as sequentially numbered containers), describing any steps taken to conceal the sequence until interventions were assigned	7	14.58

Implementation	10 Who generated the random allocation sequence, who enrolled participants, and who assigned participants to interventions	7	14.58
Blinding	11a If done, who was blinded after assignment to interventions (for example, participants, care providers, those assessing outcomes) and how	1	2.08
	11b If relevant, description of the similarity of interventions	1	2.08
Statistical methods	12a Statistical methods used to compare groups for primary and secondary outcomes	45	93.75
	12b Methods for additional analyses, such as subgroup analyses and adjusted analyses	0	0
**Results**			
Flow chart	13a For each group, the numbers of participants who were randomly assigned, received intended treatment, and were analysed for the primary outcome	48	100
	13b For each group, losses and exclusions after randomisation, together with reasons	13	27.08
Recruitment	14a Dates defining the periods of recruitment and follow-up	41	85.42
	14b Why the trial ended or was stopped	0	0
Baseline data	15 A table showing baseline demographic and clinical characteristics for each group	20	41.67
Intent-to-treat analysis	16 For each group, number of participants (denominator) included in each analysis and whether the analysis was by original assigned groups	2	4.17
Outcomes and estimation	17a For each primary and secondary outcome, results for each group, and the estimated effect size and its precision (such as 95% confidence interval)	48	100
	17b For binary outcomes, presentation of both absolute and relative effect sizes is recommended	0	0
Ancillary analyses	18 Results of any other analyses performed, including subgroup analyses and adjusted analyses, distinguishing pre-specified from exploratory	0	0
Harms	19 All important harms or unintended effects in each group (for specific guidance see CONSORT for harms)	10	20.83
**Discussion**			

Limitations	20 Trial limitations, addressing sources of potential bias, imprecision, and, if relevant, multiplicity of analyses	9	18.75
Generalisability	21 Generalisability (external validity, applicability) of the trial findings	12	25
Interpretation	22 Interpretation consistent with results, balancing benefits and harms, and considering other relevant evidence	39	81.25
**Other information**			
Registration	23 Registration number and name of trial registry	1	2.08
Protocol	24 Where the full trial protocol can be accessed, if available	1	2.08
Funding	25 Sources of funding and other support (such as supply of drugs), role of funders	27	56.25

Supplementary Table 3. Rating of overall quality using items from the STRICTA guideline (n = 48)

Criteria	Description	Number of positive trials (n)	Percentage (%)
Acupuncture rationale	(1a) Style of acupuncture (e.g., traditional Chinese medicine, Japanese, Korean, Western medical, five element, ear acupuncture, etc.)	48	100
	(1b) Reasoning for treatment provided, based on historical context, literature sources and/or consensus methods, with references where appropriate	29	60.42
	(1c) Extent to which treatment was varied	0	0
Details of needling	(2a) Number of needle insertions per subject per session (mean and range where relevant)	11	22.92
	(2b) Names (or location if no standard name) of points used (uni-/bilateral)	48	100
	(2c) Depth of insertion, based on a specified unit of measurement or on a particular tissue level	15	31.25
	(2d) Responses sought (e.g., de qi or muscle twitch response)	33	68.75
	(2e) Needle stimulation (e.g., manual or electrical)	48	100
	(2f) Needle retention time	42	87.5
	(2g) Needle type (diameter, length and manufacturer or material)	48	100
Treatment regimen	(3a) Number of treatment sessions	48	100
	(3b) Frequency and duration of treatment sessions	48	100
Other components of treatment	(4a) Details of other interventions administered to the acupuncture group (e.g., moxibustion, cupping, herbs, exercises, lifestyle advice)	16	33.33
	(4b) Setting and context of treatment, including instructions to practitioners, and information and explanations to patients	6	12.5
Practitioner background	(5) Description of participating acupuncturists (qualification or professional affiliation, years in acupuncture practice, other relevant experience)	3	6.25
Control or comparator interventions	(6a) Rationale for the control or comparator in the context of the research question, with sources that justify the choice(s)	14	29.17
	(6b) Precise description of the control or comparator. If sham acupuncture or any other type of acupuncture-like control is used, provide details as for items 1–3 above	41	85.42

Supplementary Table 4. Results of the sensitivity analyses by omitting the single study

Study ID	Comparison	MD/SMD (95% CI)	Tau^2	I^2	*P*
**MMSE**					
Hu FX 2019	MA + WM vs. WM	2.22 (1.73, 2.71)	0.09	14%	< 0.00001
Zhang H 2008	EA + WM vs. WM	0.17 (-1.22, 1.56)	0.36	25%	0.81
**HDS**					
Li Y 2009	MA vs. WM	1.41 (0.73, 2.09)	0.09	10%	< 0.0001
**MoCA**					
Qu B 2020	Acupuncture + WM vs. WM	1.85 (1.22, 2.48)	0	0%	< 0.00001
**ADAS-cog**					
Li LL 2014	Acupuncture vs. WM	-0.5 (-0.77, -0.23)	/	/	0.0003
Yang JW 2019	Acupuncture vs. WM	-1.12 (-1.55, -0.7)	/	/	< 0.00001
**ADLS**					
Li LL 2014	Acupuncture vs. WM	-1.87 (-2.91, -0.82)	0.67	50%	0.0005
**BI**					
Li PF 2012	Acupuncture vs. WM	3.88 (1.41, 6.35)	0	0%	0.002
Hu FX 2019	Acupuncture + WM vs. WM	5.17 (3.16, 7.18)	0	0%	< 0.00001
Jiang YJ 2019	Acupuncture + UC vs. UC	22.04 (18.27, 25.82)	9.35	84%	< 0.00001

ADAS-cog, Alzheimer's Disease Assessment Scale-cognitive subscale; ADLS, Activities of Daily Living Scale; BI, Barthel Index; EA, electroacupuncture; MA, manual acupuncture; MD, mean difference; SMD, standard mean difference.
