# Supplementary material for: Effectiveness and Safety of Acupuncture for Vascular Cognitive Impairment: A Systematic Review and Meta-Analysis
Source: Front Aging Neurosci. 2021 Aug 6;13:692508. doi: 10.3389/fnagi.2021.692508 (PMC8377366; doi:10.3389/fnagi.2021.692508)
Supplement: Supplementary file 2 [file Image_1.pdf]

Supplementary Material

Effectiveness and Safety of Acupuncture for Vascular Cognitive Impairment: A Systematic Review and Meta-Analysis

Xin-Tong Su[1,2], Ning Sun[3], Na Zhang[4], Li-Qiong Wang[1], Xuan Zou[1], Jin-Ling Li[1], Jing-Wen Yang[1], Guang-Xia Shi[1*] and Cun-Zhi Liu[1,2*]

[1] *International Acupuncture and Moxibustion Innovation Institute, School of Acupuncture-Moxibustion and Tuina, Beijing University of Chinese Medicine, Beijing, China*

[2] *Traditional Chinese Medicine (TCM) in the Prevention and Rehabilitation of Stroke Task Force, World Stroke Organization, Geneva, Switzerland*

[3] *Acupuncture and Tuina School/The 3rd Teaching Hospital, Chengdu University of Traditional Chinese Medicine, Chengdu, China*

[4] *School of Acupuncture-Moxibustion and Tuina, Shandong University of Chinese Medicine, Jinan, China*

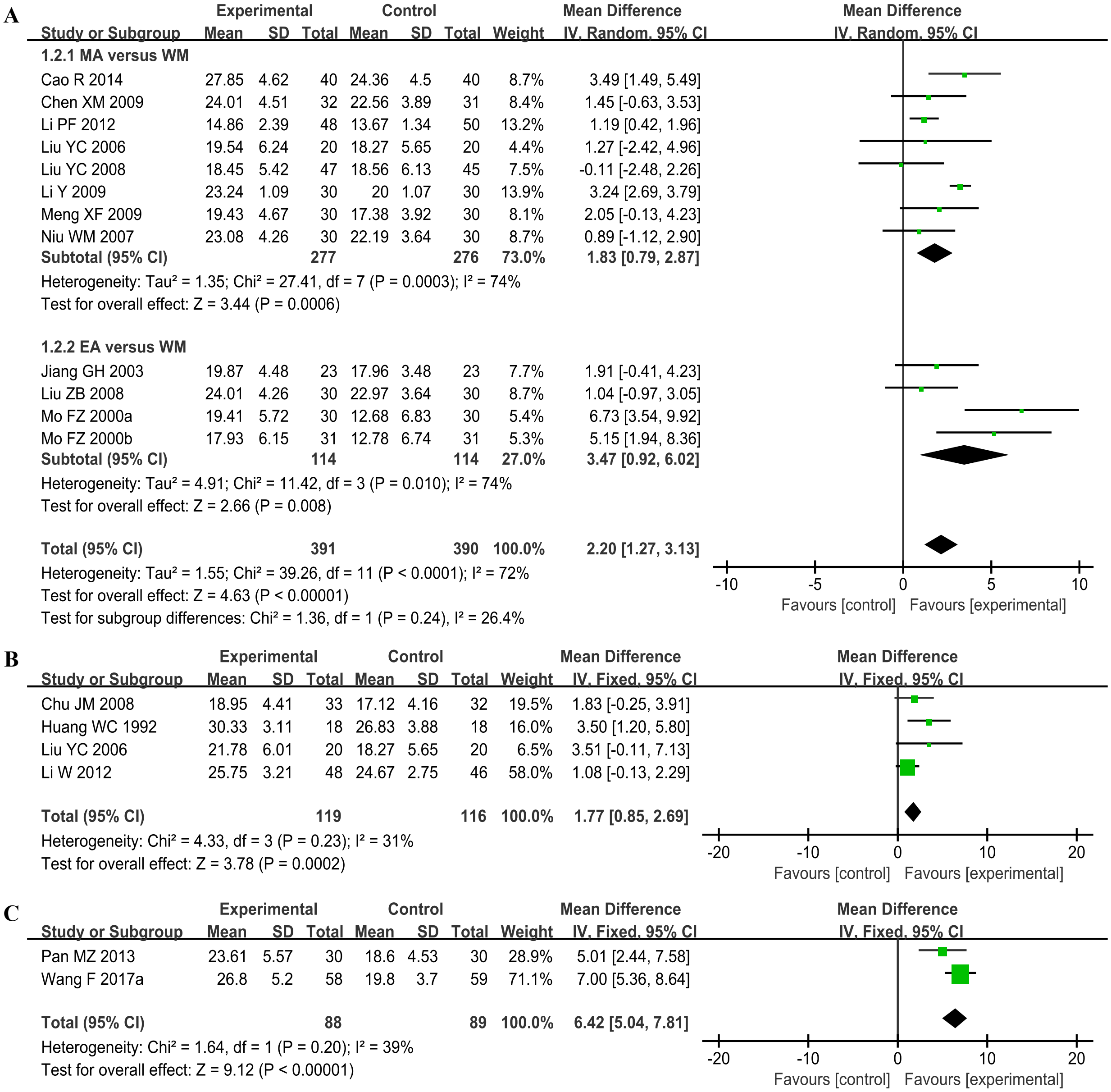

Supplementary Figure 1. The forest plot of HDS. (A) acupuncture vs. WM, (B) acupuncture plus WM vs. WM alone, (C) acupuncture plus UC vs. UC alone.

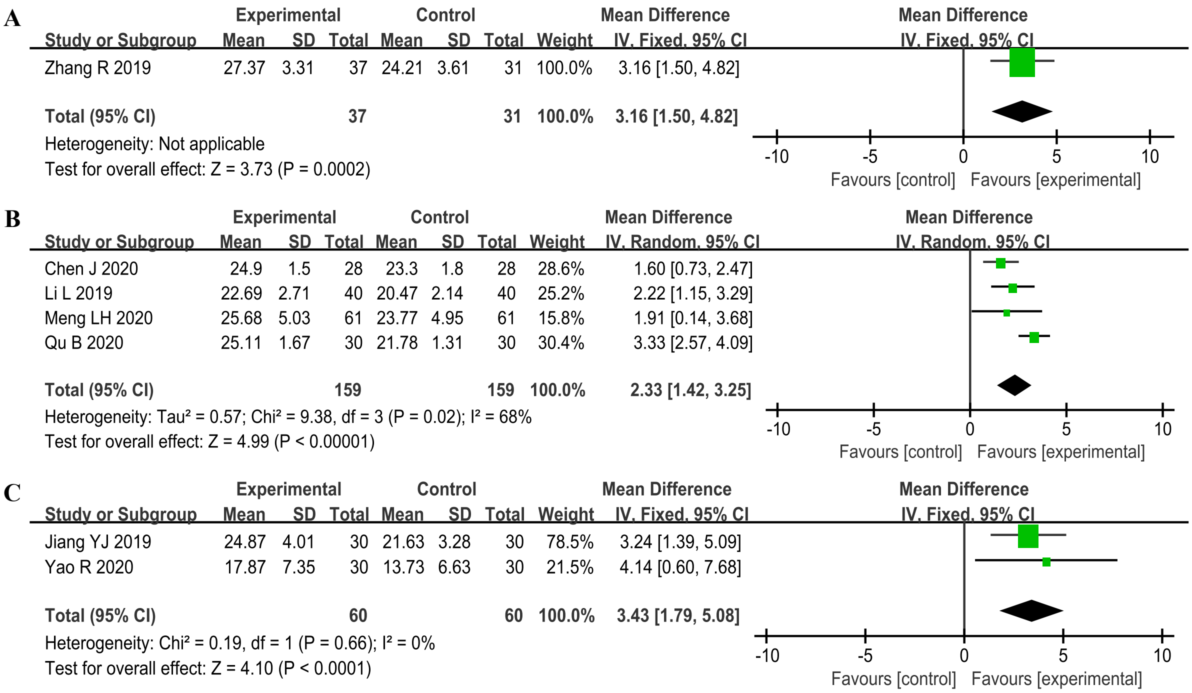

Supplementary Figure 2. The forest plot of MoCA. (A) acupuncture vs. WM, (B) acupuncture plus WM vs. WM alone, (C) acupuncture plus UC vs. UC alone.

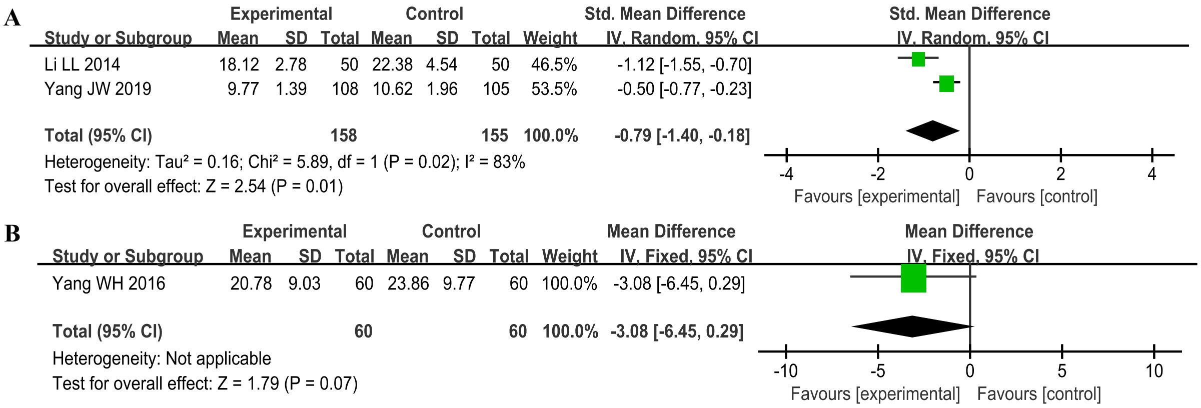

Supplementary Figure 3. The forest plot of ADAS-cog. (A) acupuncture vs. WM, (B) acupuncture plus WM vs. WM alone.

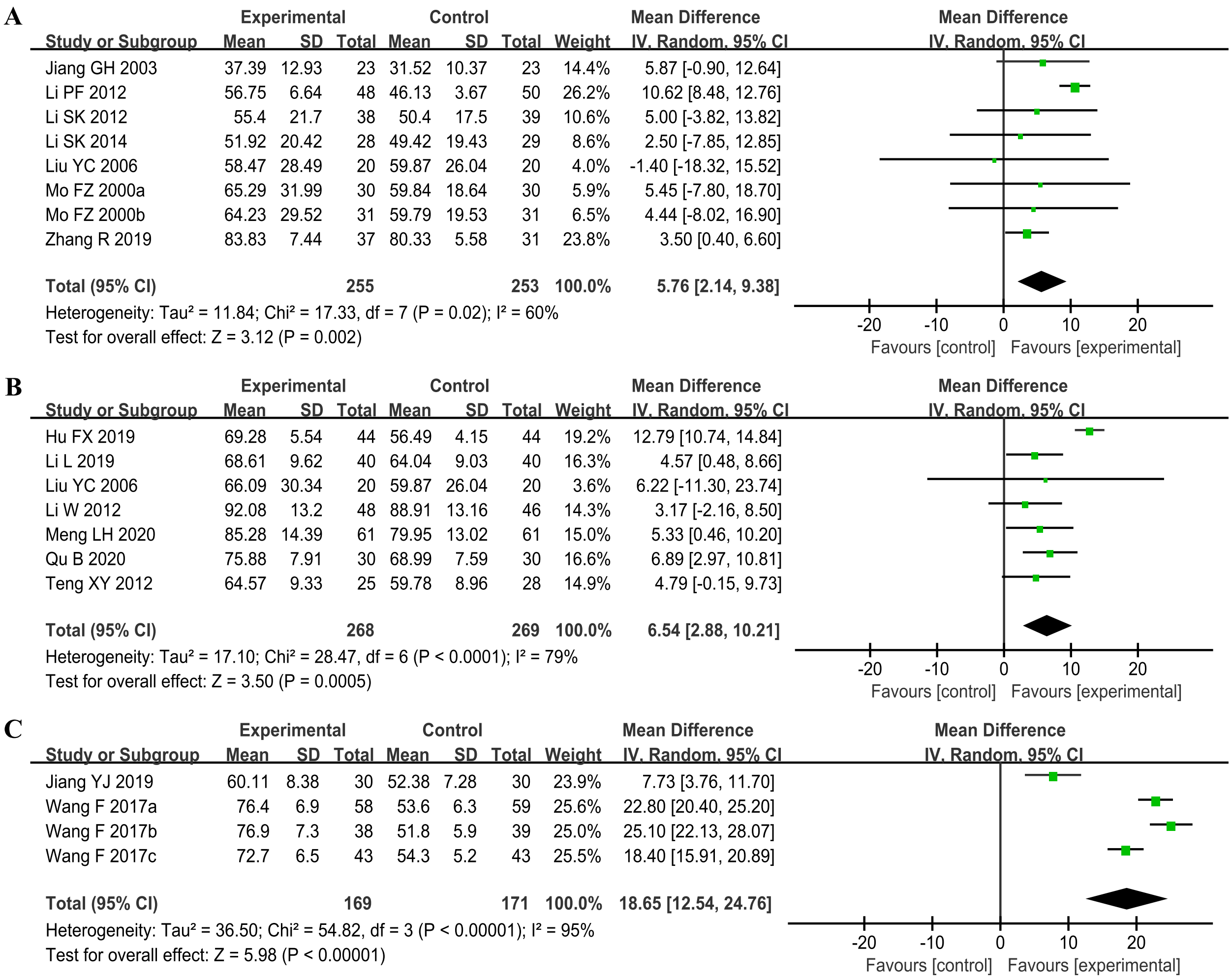

Supplementary Figure 4. The forest plot of BI. (A) acupuncture vs. WM, (B) acupuncture plus WM vs. WM alone, (C) acupuncture plus UC vs. UC alone.

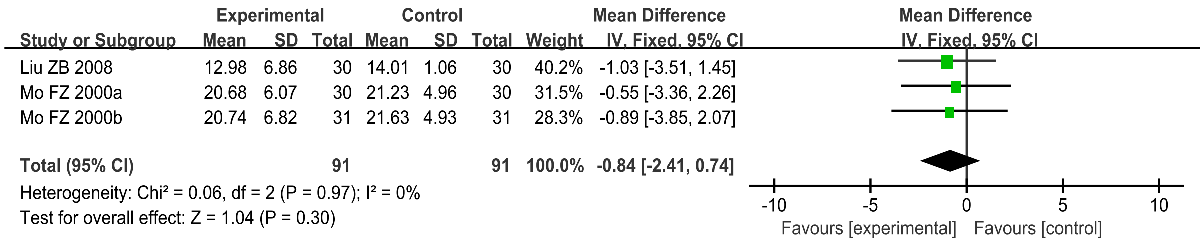

Supplementary Figure 5. The forest plot of FAQ comparing acupuncture vs. WM.

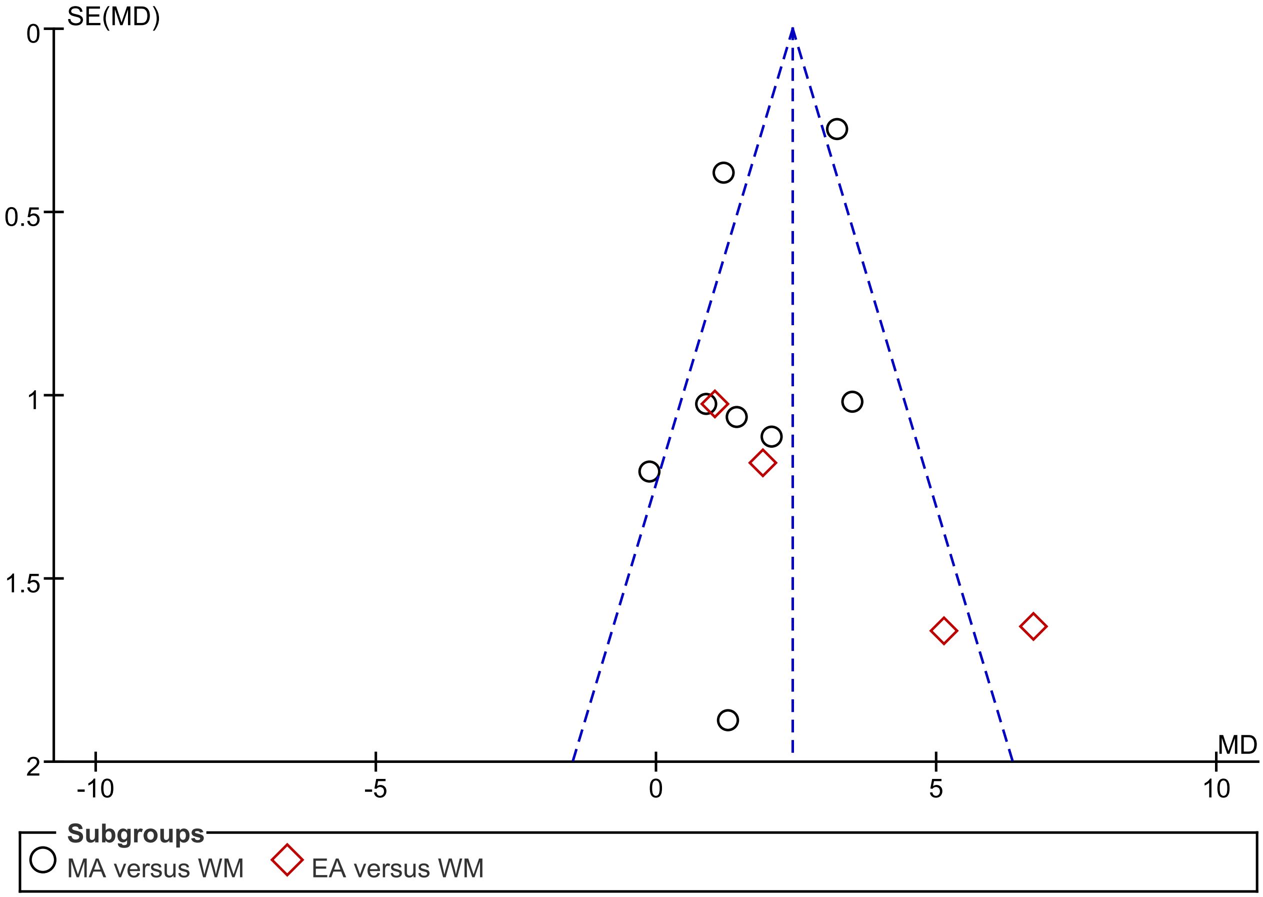

Supplementary Figure 6. The funnel plot of HDS comparing acupuncture vs. WM.
